# A self-assembling nanoplatform for pyroptosis and ferroptosis enhanced cancer photoimmunotherapy

**DOI:** 10.1038/s41377-024-01673-1

**Published:** 2025-01-02

**Authors:** Zhichao Wang, Yuqi Tang, Quan Li

**Affiliations:** 1 Institute of Advanced Materials and School of Chemistry and Chemical Engineering, Southeast University, Nanjing, 211189 China; 2 Materials Science Graduate Program, Kent State University, Kent, OH 44242 USA

**Keywords:** Biomedical materials, Biomaterials - cells

## Abstract

The microenvironment of immunosuppression and low immunogenicity of tumor cells has led to unsatisfactory therapeutic effects of the currently developed nanoplatforms. Immunogenic cell death, such as pyroptosis and ferroptosis, can efficiently boost antitumor immunity. However, the exploration of nanoplatform for dual function inducers and combined immune activators that simultaneously trigger pyroptosis and ferroptosis remains limited. Herein, a multifunctional pH-responsive theranostic nanoplatform (M@P) is designed and constructed by self-assembly of aggregation-induced emission photosensitizer MTCN-3 and immunoadjuvant Poly(I: C), which are further encapsulated in amphiphilic polymers. This nanoplatform is found to have the characteristics of cancer cell targeting, pH response, near-infrared fluorescence imaging, and lysosome targeting. Therefore, after targeting lysosomes, M@P can cause lysosome dysfunction through the generation of reactive oxygen species and heat under light irradiation, triggering pyroptosis and ferroptosis of tumor cells, achieving immunogenic cell death, and further enhancing immunotherapy through the combined effect with the immunoadjuvant Poly(I: C). The anti-tumor immunotherapy effect of M@P has been further demonstrated in in vivo antitumor experiment of 4T1 tumor-bearing mouse model with poor immunogenicity. This research would provide an impetus as well as a novel strategy for dual function inducers and combined immune activators enhanced photoimmunotherapy.

## Abstract (web summary)

A novel multifunctional nanoplatform is found to be able to induce pyroptosis and ferroptosis of tumor cells, enhancing photoimmunotherapy through the combined effect with the immunoadjuvant.

## Graphical abstract

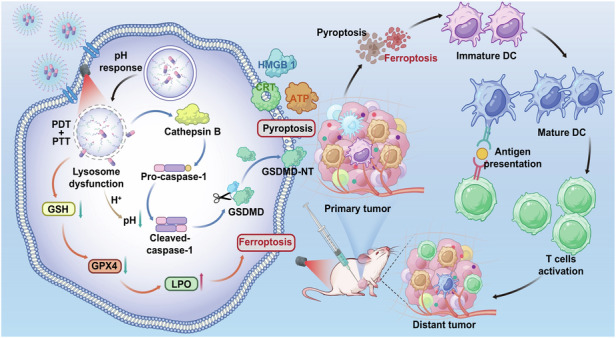

## Introduction

Immunotherapy is a novel cancer treatment with promising prospects for broad application^1^. It employs the natural immune response of the human body to combat cancer, effectively preventing metastasis and residual cancer cells, and has fewer side effects than traditional treatment methods^2,3^. However, current cancer immunotherapy still faces many limitations, including the low immunogenicity of cancer cells and the immunosuppressive tumor microenvironment^4^. Immunogenic cell death (ICD) is a unique immune response induced by programmed cell death^5^. During the process of ICD, cancer cells produce a series of signaling molecules called damage-associated molecular patterns (DAMPs), which include calreticulin (CRT) exposed on the cell surface, high mobility group box 1 (HMGB1) translocated from the nucleus to cytoplasm and adenosine triphosphate (ATP) released from the cells^6^. These DAMPs can act as natural immune adjuvants by binding to pattern recognition receptors on the surface of dendritic cells, promoting their maturation and initiating a series of cellular responses that ultimately activate innate and adaptive immune responses^7–9^. Poly(I: C) is formed by the polymerization of inosinic acid and cytidylic acid and well known as an agonist of Toll-like Receptor 3 (TLR 3) that can stimulate tumor cells to secrete immune factors, such as IL-6 and IFN-α, and then activate the tumor immune response^10,11^. However, the required high dosage and low immune response rate result in a narrow therapeutic window, significantly limiting its clinical application^12,13^. It has been demonstrated that photodynamic therapy (PDT) and photothermal therapy (PTT) induce the generation of cytotoxicity, as well as the production of tumor antigens (TAs) and the release of pro-inflammatory mediators, which can trigger immune response^14,15^. Therefore, PDT and PTT have the potential to be combined with Poly(I: C) for immune activation, compensating for the limitations of Poly(I: C) in clinical applications.

Due to the presence of apoptotic resistance in cancer cells, the therapeutic effect is often limited and unsatisfactory. Pyroptosis and ferroptosis, as two typical forms of ICD^7,8^, have been shown to activate or modulate the immune system. Pyroptosis is a novel form of lytic cell death activated by the gasdermin protein family (including gasdermin D (GSDMD) etc.)^16^. Cleaved gasdermin proteins form pores in the cell membrane, causing membrane disruption and the release of significant quantities of pro-inflammatory cytokines and intracellular contents. Ferroptosis is characterized by the depletion of glutathione (GSH), the reduction in glutathione peroxidase 4 (GPX4) activity, and the failure to metabolize lipid peroxides (LPO)^17^. In previous studies, immunotherapy mediated by pyroptosis and ferroptosis mainly relied on chemotherapeutic drugs^18,19^. However, nonspecific targeting and severe side effects of traditional drugs limit their application. Therefore, developing effective, cancer cell-targeting, and low-side-effect pyroptosis and ferroptosis inducers, especially dual function inducers, is an attractive and challenging task.

Due to the significant therapeutic effects of lysosomal cell death and its crucial role in regulating cell death, lysosomes have become one of the most promising targets for cancer therapy^20–22^. Increasing evidence suggests that lysosomal dysfunction can induce lysosomal membrane permeabilization (LMP), releasing a large number of substances such as cathepsin B and ions into the cytoplasm and triggering various kinds of ICD^23,24^. Additionally, the acidic characteristics of the lysosomal region in tumor cells are commonly utilized to regulate drug release from acid-sensitive prodrugs or pH-responsive drug carriers^25^. Currently, the mechanism of action of lysosome-targeting drugs is still ambiguous, and these drugs lack the monitoring function of drug distribution^26^. Therefore, developing an effective lysosome-targeting nanoplatform with monitoring and therapeutic functions is significant, but remains a challenging task.

Phototherapy, known for adjustable light-induced damage, non-invasiveness, and minimal side effects, is a promising interventional cancer therapy^27–29^. Two rational approaches to achieving phototherapy are PDT and PTT. In PDT and PTT, photosensitizers can generate cytotoxic reactive oxygen species (ROS) under light irradiation, while photothermal agents can convert light energy into heat efficiently. Photosensitizers targeting lysosomes can induce lysosomal dysfunction by generating ROS and heat, and subsequently trigger ICD^30–32^. However, traditional photosensitizers with planar rigid structures often undergo aggregation-caused quenching (ACQ) when in an aggregated state, thereby promoting non-radiative transitions and reducing ROS yield^33^. Aggregation-induced emission luminogens (AIEgens) have demonstrated significant advantages in fluorescence imaging and phototherapy due to their enhanced fluorescence, excellent photostability, and large Stokes shift in the aggregated state^34–37^. Therefore, designing pH-responsive lysosome-targeted nanoplatforms based on AIEgens is considered a breakthrough strategy in cancer theranostics.

**Fig. 1:**
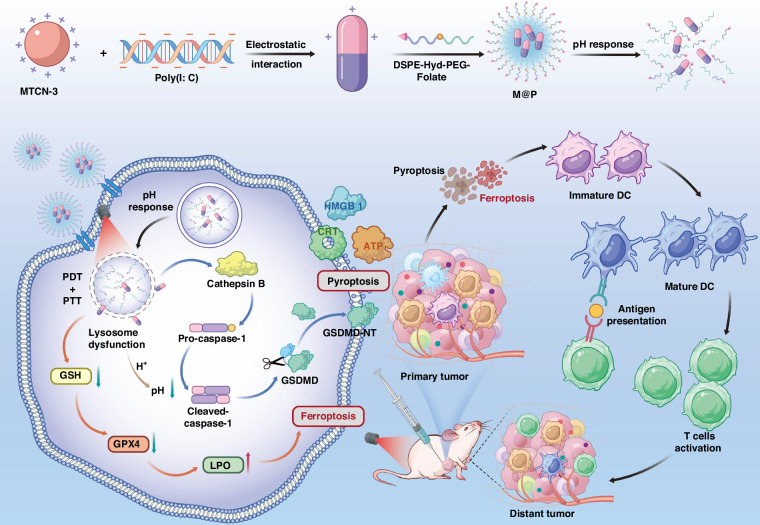
Illustration of multifunctional nanoplatforms M@P inducing cancer cells pyroptosis and ferroptosis for cancer photoimmunotherapy

Herein, we report a photoinduced pyroptosis and ferroptosis theranostic nanoplatform targeting lysosomes (Fig. 1). We first synthesize a series of AIEgens with a donor-acceptor (D-A) structure, MTCN-1, MTCN-2 and MTCN-3. Due to strong D-A interaction and the smallest singlet-triplet energy gaps, MTCN-3 exhibits near-infrared aggregation-induced emission (AIE), high ROS generation capabilities, and excellent photothermal effect. Then we self-assemble MTCN-3 and Poly (I: C), and further encapsulate MTCN-3 and Poly(I: C) in amphiphilic polymer DSPE-Hyd-PEG-Folate to construct a multifunctional nanoplatform (M@P) with cancer cell-targeting and pH-responsive properties. The M@P nanoplatform targeting lysosomes can generate a large amount of ROS and heat under light irradiation, leading to lysosomal dysfunction and subsequently triggering tumor pyroptosis and ferroptosis. Compared with treatment with a single photosensitizer, the fluorescence intensity of M@P significantly increases due to the restriction of intramolecular movement of MTCN-3 by poly (I: C). In addition, the combination of MTCN-3 and Poly (I: C) can enhance the efficacy of tumor immunotherapy. The in vivo experimental results of mice show that the M@P nanoplatform can actively target the tumor site within 6 h after intravenous injection and accumulate at the tumor site for 24 h. M@P has excellent tumor suppressive effects, and phototherapy combined with immune adjuvants can promote the release of TAs, triggering stronger anti-tumor immune responses. Thus, we have demonstrated that M@P, as a photoimmunotherapy nanoplatform combining phototherapy and immune adjuvant, can not only directly inhibit tumors, but also trigger anti-tumor immune responses. This is a novel strategy for nanoplatform that enhance photoimmunotherapy with dual-function inducers and combined immune activators.

## Results

### Molecular design, synthesis, and theoretical calculation

**Fig. 2:**
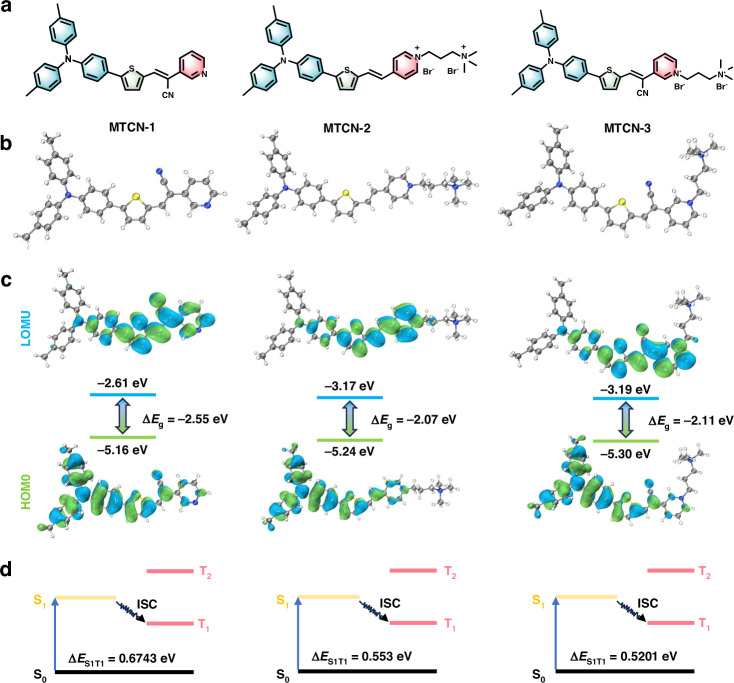
**Structure details of MTCN-1, MTCN-2, and MTCN-3. a** Chemical structures of MTCN-1, MTCN-2, and MTCN-3; **b** Optimized geometries of MTCN-1, MTCN-2, and MTCN-3; **c** Calculated HOMO-LUMO energy gaps of MTCN-1, MTCN-2 and MTCN-3; **d** Singlet and triplet energy levels and ΔE_S1T1_ values of MTCN-1, MTCN-2 and MTCN-3 determined at the M06-2X/6-311 G (d, p) level of theory, Gaussian 16 program

In this study, triphenylamine (TPA) and pyridinium were used as the electron donor (D) and acceptor (A), respectively, to construct photosensitizers with D-A structure (MTCN-1, MTCN-2, and MTCN-3) (Fig. 2a). The photosensitizer synthesis pathways were illustrated in Scheme S1. Due to the twisted conformation of TPA, it can avoid intermolecular π-π stacking, thereby enhancing the AIE effect of the aggregated state. The thiophene ring has electron-donating properties and a simple conjugated structure, which can enhance the strength of D-A and promote the intramolecular charge transfer (ICT) effect. Compared with other aromatic rings, the thiophene ring has a smaller electronic bandgap and longer absorption and emission wavelengths^38^. The cyano group further enhances the D-A intensity, promoting spatial separation of the highest occupied molecular orbital (HOMO) and lowest unoccupied molecular orbital (LUMO), leading to a reduction in the energy level difference between the singlet and triplet states. The structures of these three molecules were characterized by ^1^H NMR, ^13^C NMR, and HRMS (Fig. S1-S9). To gain deeper insights into the geometric and electronic properties of MTCN-1, MTCN-2, and MTCN-3 (Fig. 2b), time-dependent density functional theory (TD-DFT) calculations were performed at the B3LYP-D3(BJ)/def2-TZVP level to obtain optimized singlet configurations. As shown in Fig. 2c, the delocalized electron density of HOMO is primarily distributed in the triphenylamine fragment, while that of LUMO is concentrated in the pyridinium and thiophene units, indicating that the synthesized molecule has a strong ICT effect. These results were further validated by the calculated energy gaps (ΔE_g_) of MTCN-1 (-2.55 eV), MTCN-2 (-2.07 eV), and MTCN-3 (-2.11 eV). Additionally, the energy gaps between the singlet and triplet states (ΔE_S1T1_) of MTCN-1, MTCN-2, and MTCN-3 were calculated to be as low as 0.6743 eV, 0.553 eV, and 0.5201 eV, respectively (Fig. 2d), revealing their enormous potential for ROS generation. Theoretical calculations indicated that MTCN-3 exhibited the smallest ΔE_S1T1_, which is favorable for non-radiative transition formation. Additionally, due to the more active intramolecular stretching vibrations and rotations from increasing intramolecular distance between the donor and acceptor groups, it was anticipated to enhance the efficiency of photothermal conversion.

### Photophysical properties

**Fig. 3:**
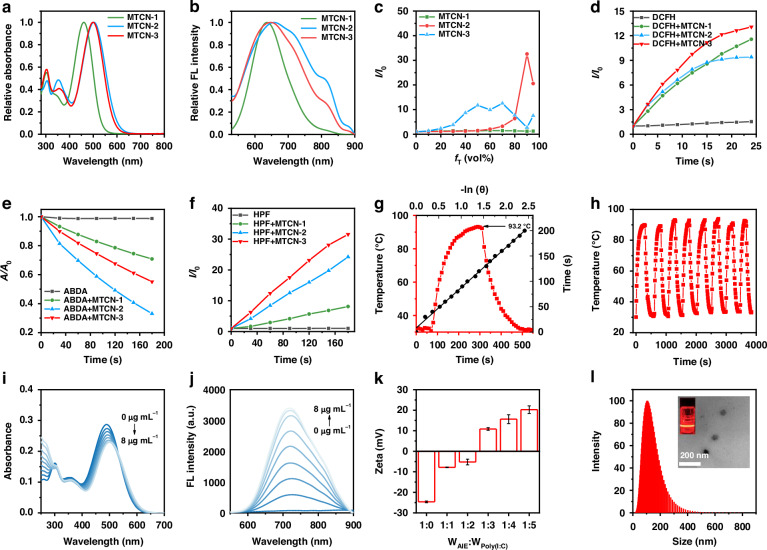
**Photophysical properties of photosensitizers and characterization of M@P. a** UV-Vis absorption spectra of MTCN-1, MTCN-2, or MTCN-3 (10 µM) in DMSO; **b** Fluorescence emission spectra of MTCN-1, MTCN-2, or MTCN-3 (10 µM) in DMSO; **c** Plot of the relative emission intensity (*I/I*_*0*_) vs toluene fraction (*f*_T_), where *I*_*0*_ = fluorescence intensity in pure DMSO; **d** ROS generation in the presence of MTCN-1, MTCN-2, or MTCN-3 (10 µM), respectively, using DCFH-DA as an indicator; **e** ^1^O_2_ generation in the presence of MTCN-1, MTCN-2, or MTCN-3 (10 µM), respectively, using ABDA as an indicator; **f** •OH generation in the presence of MTCN-1, MTCN-2, or MTCN-3 (10 µM), respectively, using HPF as an indicator; **g** Temperature elevation of MTCN-3 (100 µM) in DMSO and the plot of time versus -ln (θ) of MTCN-3. θ is the driving force of temperature; **h** Photothermal stability study of MTCN-3 during eight circles of heating-cooling processes under 520 nm laser irradiation (0.5 W cm^-2^); **i** The UV-Vis absorption and **j** fluorescence spectra of MTCN-3 (10 µg mL^-1^) following the addition of varying concentrations of Poly(I: C); **k** The optimization of the MTCN-3 to Poly(I : C) weight ratio in the self-assembly complex of MTCN-3/Poly(I : C); **l** Particle size distribution of M@P measured by DLS and TEM images

Subsequently, the optical properties of MTCN-1, MTCN-2, and MTCN-3 were investigated using ultraviolet-visible (UV-Vis) absorption and fluorescence spectroscopy. As shown in Fig. 3a, their maximum absorptions in dimethyl sulfoxide (DMSO) were 461 nm, 503 nm, and 499 nm, respectively. MTCN-3 exhibited a broad absorption range of 410-630 nm, with high absorptivity at wavelengths such as 520 nm. The maximum emission peaks of MTCN-1, MTCN-2, and MTCN-3 in DMSO were located at 636 nm, 658 nm, and 647 nm, respectively (Fig. 3b). Additionally, MTCN-2 and MTCN-3 exhibited significant AIE properties in the poor solvent, toluene (Figs. 3c and S10). The fluorescence intensity of MTCN-2 and MTCN-3 significantly increased with the increase in the toluene fraction, solidly manifesting their typical AIE properties. When the toluene fraction reached 90%, the fluorescence intensity of MTCN-2 was more 30 times higher than that in pure DMSO. The fluorescence intensity of MTCN-3 reached its maximum at the 70% toluene fraction, which was 10 times higher than in pure DMSO. The fluorescence intensity of MTCN-1 did not significantly change with the increase in the toluene fraction. We further investigated the absorption spectra of MTCN-1, MTCN-2, and MTCN-3 in different polar solvents (Fig. S11). The results indicated that with the increase of solvent polarity, the absorption spectrum exhibited a red shift, which could be attributed to the enhanced ICT effect^39^.

To validate the potential synergistic phototherapeutic effects of the molecules under 520 nm laser irradiation, we initially investigated the absorption and emission properties of MTCN-1, MTCN-2, and MTCN-3 in aqueous solution (Fig. S12). The maximum absorption of MTCN-1, MTCN-2, and MTCN-3 was observed at 455 nm, 489 nm, and 489 nm, respectively, while the maximum emission was recorded at 631 nm, 722 nm, and 722 nm, respectively. These results indicated that even in aqueous solution, the photosensitizer exhibited significant absorption at 520 nm, so we will conduct subsequent in vivo and in vitro experiments under laser irradiation at 520 nm.

To evaluate the PDT efficacy, we employed 2′,7′-dichlorodihydrofluorescein diacetate (DCFH-DA) as an indicator to assess the overall ROS generation ability (Figs. 3d and S13). As illustrated in Fig. 3d, these three photosensitizers demonstrated significant differences in both the rate and efficiency of ROS generation. It was worth noting that MTCN-3 exhibited the highest ROS production ability under white light irradiation, and the fluorescence intensity of DCFH increased by nearly 13-fold within 30 s. In comparison, the fluorescence intensity of DCFH increased by approximately 12-fold and 9-fold in the presence of MTCN-1 and MTCN-2, respectively, after 30 s of white light exposure. In addition, to further investigate the types of ROS generated by the three photosensitizers, such as singlet oxygen (^1^O_2_), hydroxyl radicals (•OH), and superoxide anions (O_2_^•−^), various ROS indicators were used, such as 9,10-anthracenediyl-bis(methylene)dimalonic acid (ABDA, an indicator of ^1^O_2_), hydroxyphenyl fluorescein (HPF, an indicator of •OH), and dihydrorhodamine 123 (DHR 123, an indicator of O_2_^•−^) (Figs. 3e-f and S14-16). The experimental results indicated that compared to other photosensitizers, MTCN-3 exhibited significant •OH generation ability. Subsequently, we investigated the photothermal behavior of MTCN-3 under 520 nm laser irradiation (Fig. 3g). MTCN-3 exhibited a significant photothermal effect in DMSO solution, reaching a maximum temperature of 93.4 °C after 300 s of irradiation. MTCN-3 demonstrated excellent photothermal stability, without any decrease observed after eight heating-cooling cycles (Fig. 3h), and the calculated photothermal conversion efficiency of MTCN-3 was 24.02%. The excellent ROS generation and photothermal properties of MTCN-3 demonstrated its potential in PDT and PTT applications.

MTCN-3 was selected as the subject for subsequent experiments due to its excellent ROS generation ability and photothermal effect. Considering the advantage of enhanced permeability and retention effect of nanoparticles in anti-tumor therapy, we constructed a self-assembled nanoplatform of MTCN-3 and Poly(I: C) for in vivo monitoring and therapy. Poly(I: C) is an artificial immune adjuvant that can further enhance the efficacy of phototherapy in tumor immunotherapy^10,11^. The negatively charged Poly(I: C) could spontaneously self-assemble with the positively charged MTCN-3 to form a complex. We investigated the self-assembly characteristics of MTCN-3 and Poly(I: C) by changing the concentrations of Poly(I: C) (Fig. 3i-j). With the addition of Poly(I: C), the emission of MTCN-3 was gradually enhanced at 722 nm, reaching its maximum intensity at 7 µg mL^-1^. The fluorescence was enhanced 44-fold (Fig. S17), which proved that Poly(I: C) facilitated the aggregation of MTCN-3 in aqueous solutions via electrostatic self-assembly. We further investigated the optimal self-assembly ratio of MTCN-3 and Poly (I: C), and validated it through zeta potential measurements (Fig. 3k). When the weight ratio of MTCN-3 to Poly(I: C) exceeded 3: 1, Poly(I: C) could electrostatically bind as much MTCN-3 as possible, resulting in a slightly positive charge. To further enhance the biocompatibility and tumor-targeting ability of the nanoparticles, an amphiphilic shell containing targeting moieties (folate group) and pH-responsive bonds (hydrazone) was used to encapsulate the electrostatically self-assembled nanoparticles, forming a nanoplatform M@P. The average particle size of M@P was about 103.4 nm according to dynamic light scattering (DLS), consistent with the transmission electron microscopy (TEM) images (Fig. 3l). The zeta potential of the M@P nanoparticles had a positive value of 18.85 ± 1.66 mV. Notably, after one week of storage at room temperature in an aqueous solution or phosphate-buffered saline (PBS), the nanoscale changes of M@P could be ignored (Fig. S19a), indicating that M@P had high stability. It is well known that the physiological environment is weakly basic (pH 7.4), whereas the lysosome is an acidic organelle (pH 5.0 or lower). Based on this, we investigated the pH-responsive behavior of the M@P nanoparticles at 37 °C in PBS (pH 7.4) and acetate-buffered saline (ABS) (pH 5.0). In ABS (pH 5.0), TEM images showed noticeable disintegration of the nanoparticles (Fig. S18), demonstrating that M@P had pH-responsive characteristics. The photothermal effect of M@P with different concentrations was also investigated using a 520 nm laser, and the experimental results indicated that MTCN-3 maintained high photothermal performance after being encapsulated as M@P (Fig. S19b).

### Cellular uptake, lysosome targeting and cytotoxicity

**Fig. 4:**
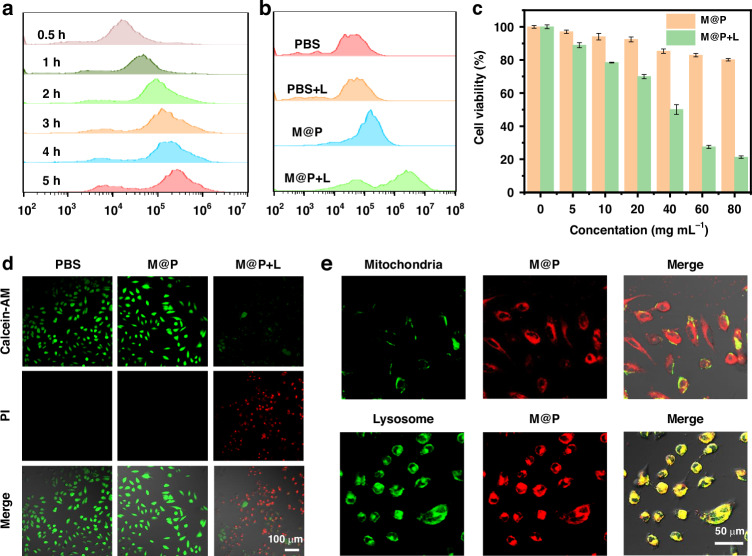
**Cellular uptake, lysosome targeting and cytotoxicity. a** Flow cytometry of MDA-MB-231 cells upon incubation with M@P; **b** Flow cytometry of intracellular ROS generation; **c** Cell viability of MDA-MB-231 cells with different concentrations of M@P (L: 520 nm light, 0.5 W cm^-2^, 1 min); **d** Live/dead staining of MDA-MB-231 cells with M@P (L: 520 nm light, 0.5 W cm^-2^); **e** CLSM images of MDA-MB-231 cells stained with M@P and their co-staining with MitoTracker Green and LysoTracker Green (1 μM)

We used flow cytometry to investigate the cellular uptake of M@P (Fig. 4a). The experimental results indicated that within 4 h of incubation, M@P could be uptaken into MDA-MB-231 cells. We further validated it through a confocal laser scanning microscope (CLSM), and the CLSM images also showed similar results, demonstrating that M@P could be uptaken into cells within 4 h (Fig. S20). Subsequently, we evaluated the intracellular ROS generation ability of M@P under light irradiation (M@P + L) by flow cytometry and CLSM (Figs. 4b and S21). The experimental results showed a significant increase in fluorescence signal in the M@P + L group, indicating that M@P could generate a large amount of ROS. Additionally, the intracellular generation of various types of ROS was assessed using singlet oxygen sensor green fluorescent probe (SOSG), HPF, and DHR123. The experimental results showed that M@P could not only generate type-II ROS (^1^O_2_), but also generated a large amount of type-I ROS (O_2_^•−^ and •OH), which was consistent with the above extracellular test results (Fig. S22). The cytotoxicity of M@P on MDA-MB-231 cells was measured using the MTT (3-(4,5-dimethylthiazol-2-yl)-2,5-diphenyltetrazolium bromide) assay (Fig. 4c). M@P exhibited negligible cytotoxicity to MDA-MB-231 cells under dark conditions, even at concentrations up to 80 μg mL^-1^, indicating that M@P had preferably biocompatibility. However, after 520 nm laser irradiation (0.5 W cm^-2^) for 1 min, the cell viability significantly decreased with increasing M@P concentration, indicating that M@P had a significant phototoxicity on MDA-MB-231 cells. To directly demonstrate the antitumor efficacy of M@P, live/dead cell co-staining experiments were performed using calcein-AM (a living cell staining agent) and propidium iodide (PI, a dead cell staining agent) (Fig. 4d). In the control group, only green fluorescence signals were observed, indicating that the cytotoxicity of M@P in the absence of light could be ignored. In contrast, strong red fluorescence signals were observed in the M@P + L group, demonstrating that M@P could effectively kill tumor cells under light irradiation. Next, we investigated the subcellular distribution of M@P in MDA-MB-231 cells. As shown in Fig. 4e, when co-staining with LysoTracker-Green (a lysosome staining agent targeting the lysosome), the red fluorescence signal of M@P overlapped well with the green fluorescence signal of LysoTracker-Green, with a Pearson correlation coefficient of 0.84, indicating the excellent lysosome-targeting ability of M@P. Meanwhile, we also ruled out the possibility of targeting other organelles (Fig. S23).

### Inducing pyroptosis, ferroptosis and immunogenic cell death

**Fig. 5:**
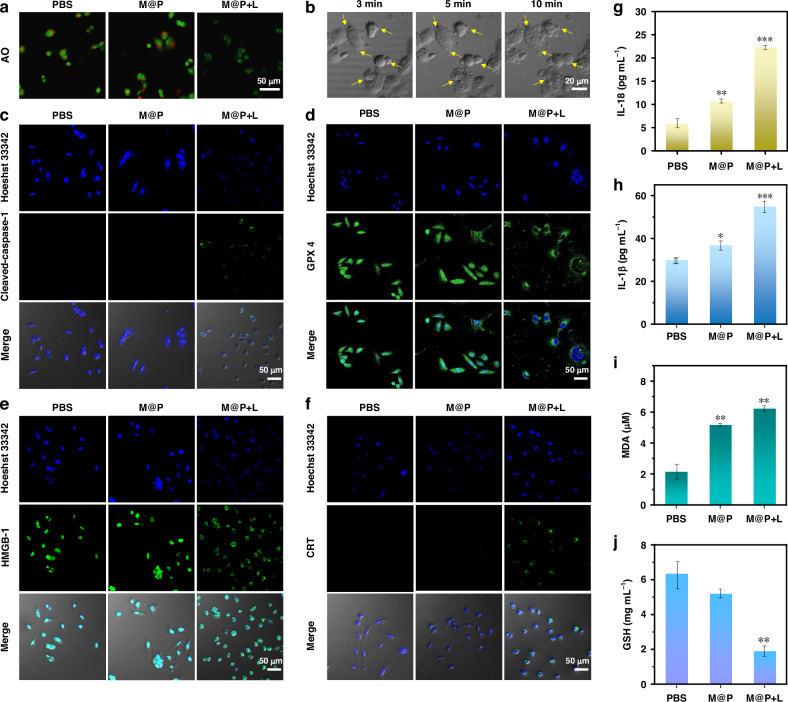
**Tumor cell pyroptosis, ferroptosis and immunogenic cell death. a** CLSM images of AO-stained MDA-MB-231 cells incubated with M@P (L: 520 nm light, 0.5 W cm^-2^, 5 min); **b** Real-time observation of the pyroptosis process; Immunofluorescent staining of **c** Cleaved-caspase-1, **d** GPX-4, **e** CRT and **f** HMGB-1 activation in MDA-MB-231 cells; A Quantitative analysis of secretion of **g** IL-18 and **h** IL-1β in MDA-MB-231 cells treated with M@P, measured by ELISA; Quantitative analysis of **i** MDA and **j** GSH in MDA-MB-231 cells treated with M@P (L: 520 nm light, 0.5 W cm^-2^, 5 min). Mean ± SD, n = 3, *p < 0.05, **p < 0.01, ***p < 0.001

To investigate whether M@P treatment could cause lysosomal dysfunction, we used acridine orange (AO) as an indicator to evaluate lysosome integrity (AO could emit red fluorescence in acidic lysosomes and green fluorescence in cytoplasm and nucleus) (Figs. 5a and S24). As shown in Fig. 5a, after treatment with M@P + L, the red fluorescence signals of AO completely disappeared in MDA-MB-231 cells, indicating that M@P + L could induce lysosomal dysfunction. The occurrence of lysosomal dysfunction, releases the protons from the lysosome into the cytoplasm, triggering intracellular acidification, which is detrimental to the cells^40^. Therefore, we measured the intracellular pH of cancer cells by using a commercial pH indicator (BCECF-AM). As shown in Fig. S25, strong green fluorescence signals were observed in cells incubated with M@P or PBS, but almost no green fluorescence signal in cells incubated with M@P under 520 nm laser irradiation, which indicated the intracellular pH after treatment with M@P + L appreciably decreased. We focused on studying the downstream signaling pathways of lysosome rupture due to the intimate relationship between lysosome dysfunction and ICD. The rupture of lysosomes may trigger variety of subtypes of regulated cell death, such as pyroptosis and ferroptosis^23,24^. Firstly, we observed the morphological changes in cells treated with M@P using CLSM (Figs. 5b and S26). When M@P-treated cancer cells were irradiated with a 520 nm laser, the cells swelled and the plasma membrane gradually expanded, demonstrating an obvious pyroptosis process. Subsequently, we continued to explore how lysosome rupture induces pyroptosis. According to the pervious study, damaged lysosomes can release hydrolytic enzymes, cathepsin B, which can induce pyroptosis^23^. So, we observed the activity of cathepsin B in cancer cells using immunofluorescence staining (Fig. S27). From the CLSM images, it was shown that cathepsin B in the M@P + L group was released from lysosomes into the cytoplasm due to lysosome rupture. Cathepsin B cleaves pro-caspase-1 to form cleaved-caspase-1, inducing pyroptosis^41^, and we confirmed the notable upregulation of cleaved-caspase-1 expression through CLSM (Fig. 5c). In pyroptosis, cleaved-caspase-1 will cleave GSDMD to the N-terminal of GSDMD (GSDMD-NT)^42^; GSDMD-NT forms membrane pores and triggers membrane rupture (Fig. S28a). Additionally, IL-18, IL-1β, and LDH were released to the extracellular environment, thereby activating anti-tumor immune responses (Figs. 5g-h and S28b).

The accumulation of intracellular ROS and lipid peroxidation (LPO) were regarded as important hallmarks of ferroptosis. To determine whether lysosomal dysfunction triggered by M@P contributes to ferroptosis, we conducted further research. M@P could generate a significant amount of cellular ROS, which is beneficial for the occurrence of ferroptosis^17^. Subsequently, we used the MDA lipid peroxidation assay kit to investigate lipid peroxide levels. The result showed that M@P significantly enhanced intracellular MDA levels under 520 nm laser irradiation, indicating significant accumulation of lipid peroxides (Fig. 5i). In addition, CLSM imaging revealed a significant downregulation of GPX4 content in M@P + L treated cells, which is another key characteristic of ferroptosis (Fig. 5d), indicating that a large amount of lipid peroxidation during ferroptosis could deplete GPX4. At the same time, we also measured the content of glutathione (GSH), an important substrate of GPX4. The experiment found that when cells were treated with M@P + L, the expression of GSH was significantly decreased (Fig. 5j). The above results indicated that M@P could trigger lysosomal dysfunction, further inducing pyroptosis and ferroptosis.

Pyroptosis and ferroptosis are two typical ICD forms that can induce acute inflammatory responses, thereby activating anti-tumor immune responses^17^. To evaluate the effect of M@P + L induced ICD, we detected ICD markers (CRT, HMGB-1, and ATP). As shown in Fig. 5e and f, M@P + L could significantly promote the exposure of CRT on the cell surface and the migration of HMGB-1 from the nucleus to the entire cell. In addition, we detected the level of extracellular ATP and found a significant increase in ATP content (Fig. S28c). These experimental results demonstrated that M@P could induce pyroptosis and ferroptosis through phototherapy, thereby activating a strong anti-tumor immune response. Next, we used the transwell assay to investigate whether tumor cells undergoing ICD could release TAs (Fig. S29). We seeded RAW 264.7 cells in the upper chamber and added the supernatant to the lower chamber. The migration of cells from the upper chamber to the lower chamber demonstrated that the TAs released by ICD significantly promoted the migration of RAW 264.7 cells.

### Therapeutic effects and immunotherapy in vivo

**Fig. 6:**
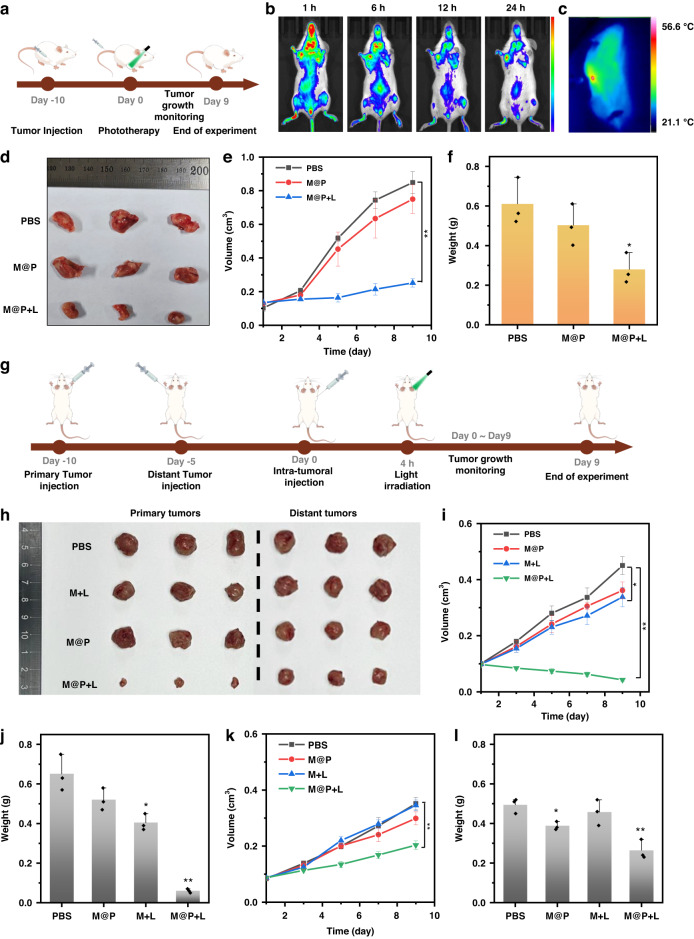
**Anti-tumor effect of M@P in vivo. a** The treatment process of unilateral 4T1-tumor-bearing mice; **b** In vivo fluorescence images of the 4T1-tumor-bearing mice after intravenous injection of M@P; **c** Thermal imaging of tumor region under 520 nm light (0.5 W cm^-2^); **d** Photograph of tumors in unilateral 4T1-tumor-bearing mice with different treatments; **e** Tumor volumes and **f** tumor weights of unilateral 4T1-tumor-bearing mice with different treatments; **g** The treatment process of bilateral 4T1-tumor-bearing mice; **h** Photograph of primary and distant tumors in bilateral 4T1-tumor-bearing mice with different treatments; **i** Primary tumor volumes and **j** tumor weights of mice with different treatments; **k** Distant tumor volumes and **l** tumor weights of mice with different treatments. Mean ± SD, n = 3, *p < 0.05, **p < 0.01, ***p < 0.001

Given the excellent antitumor performance of M@P in vitro, we established a 4T1 tumor-bearing mouse model to evaluate the therapeutic effects of M@P in vivo (Fig. 6a). Initially, we assessed the tumor-targeting capability of M@P in vivo. When the tumor volume in 4T1 tumor-bearing mice reached 100 mm^3^, M@P was intravenously injected into mice (10 mg kg^-1^). Fluorescence distribution in the body was monitored at different time points. In vivo, fluorescence imaging indicated that M@P could accumulate in the tumor site within 6 h, and there was still strong fluorescence at the tumor site 24 h post-injection (Fig. 6b), indicating that M@P had the ability to target tumor cells and had an enhanced permeability and retention effect at the tumor site. Then, we investigated the photothermal effect of M@P in vivo using intratumoral injection (Fig. 6c). When the M@P-accumulated tumor site was irradiated with laser, the temperature of this site quickly reached 56.6 °C within 1 min, indicating that M@P had an excellent photothermal effect.

Encouraged by the excellent fluorescence imaging and photothermal effect of M@P in vivo, we investigated the antitumor efficacy of M@P in vivo. Unilateral 4T1 tumor-bearing mice with tumors approximately 100 mm^3^ were randomly divided into three groups (PBS, M@P, M@P + L). After injecting M@P for 6 h every day, the tumor site was exposed to light (520 nm, 0.5 W cm^-2^, 5 min), and the mouse body weight and tumor volume were measured every 2 days. Compared with PBS and M@P groups, the tumor growth in the M@P + L group was significantly reduced, with a tumor growth inhibition rate of 71.89% (Fig. 6d-f), indicating that M@P had a strong anti-tumor effect under light irradiation. In addition, there was no significant difference in body weight between the three treatment groups and the control group (Fig. S30a), indicating excellent biocompatibility of M@P.

To further explore the anti-tumor photoimmunotherapy effect of M@P, a bilateral 4T1 tumor-bearing mouse model was established by inoculating 4T1 cells into both the left and right axillae of mice (Fig. 6g). Only the primary tumors on the right side were treated and the growth of the primary and distant tumors were monitored. The M@P + L group demonstrated the most significant inhibition of tumor growth, with the tumor growth inhibition rates of primary and distant tumor reaching 90.26% and 44.55%, respectively (Fig. 6h-l). The primary and distant tumors in the M@P + L group were significantly inhibited compared to the PBS group. The M@P and M + L groups exhibited slight inhibition of primary tumor growth, but had almost no inhibition of distant tumor growth. This result could be attributed to the enhanced immune activation induced by the combined effect of ICD and Poly(I: C). Notably, a steady increase in body weight was observed in all groups of mice during the experimental period, indicating the biosafety of M@P in vivo (Fig. S30b). To further investigate the antitumor mechanism of M@P, hematoxylin and eosin (H&E) staining was performed on tumor tissues and mouse organs (Fig. S31-S32). The experimental results indicated that tumor tissues in the PBS and M@P groups were dense and the cell morphology was normal. In contrast, the tumor tissues of the M@P + L and M + L groups were sparse, exhibiting extensive nuclear loss and pyknosis. Additionally, terminal deoxynucleotidyl transferase dUTP nick end labeling (TUNEL) staining showed that the M@P + L group had the highest number of cell deaths, with a few dead cells observed in the M@P and M + L groups, while the PBS group exhibited almost no cell death (Fig. S31). Subsequently, we observed the proliferation of tumor cells by measuring the expression of Ki-67 in the primary tumor. The experiment showed that tumor cells in the M@P + L group had almost lost proliferative ability (Fig. S33). Immunofluorescence staining for cleaved-caspase-1 and GSDMD was employed to assess the killing effect of M@P on tumors (Fig. S33), confirming that M@P could promote pyroptosis in tumor cells. The above experiments indicated that M@P could effectively inhibit tumor cell proliferation through photoimmunotherapy.

**Fig. 7:**
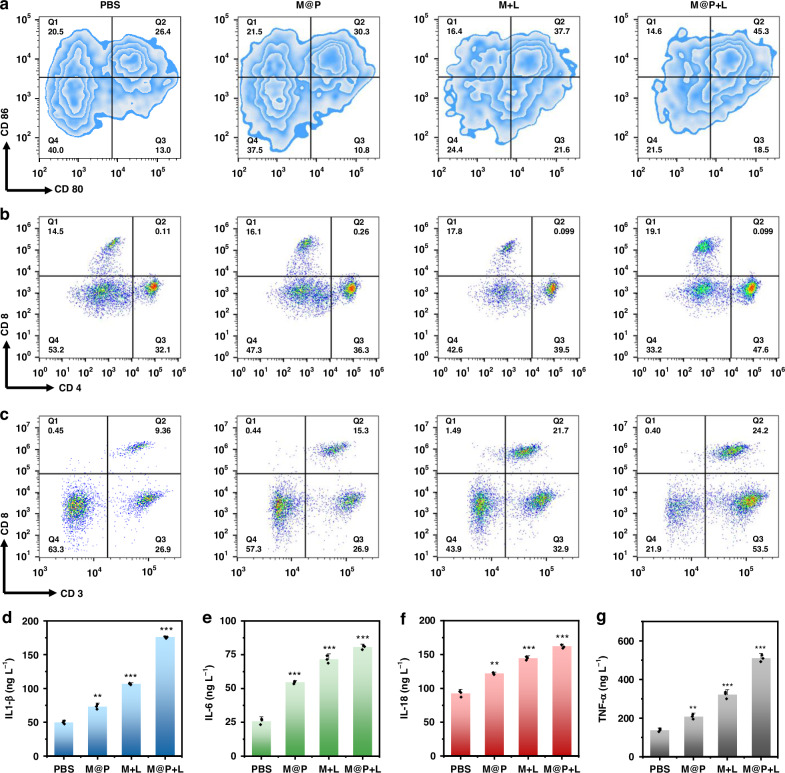
**Immunotherapy process of M@P in vivo. a** Flow cytometry analysis of mDCs (CD80^+^ and CD86^+^ gated on CD11c^+^) in primary tumors; **b** Flow cytometry analysis of primary tumor-infiltrating T lymphocytes (CD8^+^ and CD4^+^ gated on CD3^+^); **c** Flow cytometry analysis of distant tumor-infiltrating T lymphocytes (CD3^+^ and CD8^+^); Quantitative analysis of secretion of **d** IL-1β, **e** IL-6, **f** IL-18, and **g** TNF-α in serum. Mean ± SD, n = 3, *p < 0.05, **p < 0.01, ***p < 0.001

To further explore the immune mechanisms induced by M@P in vivo, immunological analyses of the primary tumor were conducted. We analyzed the markers (CD80 and CD86) of mature dendritic cells (mDCs) using flow cytometry (Fig. 7a), and the results showed that the proportion of CD80^+^ and CD86^+^ double-positive cells increased from 26.4% to 45.3%, indicating that M@P could activate systemic immune response under light irradiation. T lymphocytes are the key to destroying tumors, and their specificity for TAs is crucial. CD8^+^ T cells play a significant role in tumor antigen presentation, while CD4^+^ T cells are also essential for supporting the proper function of CD8^+^ T cells^43^. As illustrated in Fig. 7b, CD4^+^ T cells and CD8^+^ T cells were activated efficiently in the M@P + L group. The CD8^+^ T cell population in the primary tumor increased to 14.0%, and the CD4^+^ T cell population increased to 40.8% (Fig. S34). According to Fig. 7c, the proportion of CD8^+^ T cells in distant tumors increased from 9.36% to 24.2%, indicating that systemic immune response could be activated after primary tumor treatment. Simultaneously, we used ELISA assays to detect the levels of pro-inflammatory cytokines (including interleukin-6 (IL-6), IL-18, interleukin-1α (IL-1α), IL-1β, tumor necrosis factor-*α* (TNF-*α*), and interferon-*γ* (IFN-*γ*)) in serum, the results showed that the levels of pro-inflammatory cytokines in M@P + L group were significantly increased (Figs. 7d-g and S35). Additionally, the proportions of CD4^+^ and CD8^+^ T cells in the peripheral blood of mice were also elevated, which further confirmed that M@P + L could activate the systemic anti-tumor immune response (Fig. S36).

## Discussion

In this study, we have successfully designed and constructed a lysosome-targeted photoinduced pyroptosis and ferroptosis theranostic nanoplatform M@P by self-assembly of AIE photosensitizer MTCN-3 and immunoadjuvant Poly(I: C), which is further encapsulated in amphiphilic polymers. This nanoplatform can actively target tumor regions and then accumulate in the lysosomes of tumor cells, causing lysosome dysfunction through the generation of ROS and heat under light irradiation, triggering pyroptosis and ferroptosis of tumor cells, achieving immunogenic cell death, and further enhancing immunotherapy through the combined effect with the immunoadjuvant Poly(I: C). The immunotherapeutic efficacy of M@P is further demonstrated in a 4T1 tumor-bearing mouse model with poor immunogenicity. On the ninth day of treatment, the growth of primary and distant tumors in mice is effectively inhibited. The research that we disclose here provides a novel strategy for pyroptosis and ferroptosis dual function inducer design, which will benefit further advancement in the field of cancer photoimmunotherapy.

## Materials and methods

### Materials

All experimental chemical reagents were purchased from relevant chemical reagent companies that met the national standards, and no further purification was required during use, including Co. Keygen Biotechnology (including MTT, Hoechst 33342, fetal bovine serum (FBS), PBS, Annexin V-FITC/ PI apoptosis assay kit, Dulbecco’s Modified Eagle Medium (DMEM), and mouse breast tumor cells (4T1), Co. Jiangsu Meimian Industrial (all ELISA kit)), Co. Beyotime Biotechnology (including CRT Rabbit Monoclonal antibody, LDH cytotoxicity assay kit, HMGB1 Rabbit Polyclonal antibody), Co. Abcam (including cleaved-caspase-1 Rabbit Monoclonal antibody, and Anti-GSDMD antibody).

### Characterization

^1^H NMR and ^13^C NMR spectra were measured on a Bruker HW600 MHz (AVANCE AV-600) spectrometer. Electrospray ionization mass spectra (ESI-MS) were measured by Thermo Scientific Q Exactive. Record the steady-state fluorescence spectrum in the conventional quartz cell (light path 10 mm) of Shimadzu RF-6000 and Hitachi F-4700 fluorescence spectrophotometer. Record the UV-Vis spectrum in the quartz cell (light path 10 mm) of the Shimadzu UV-2700 spectrophotometer. TEM images were acquired using the JEOL JEM F200 transmission electron microscope operating at an accelerating voltage of 200 kV. CLSM images were performed on Olympus Fv3000 confocal microscopy (Olympus, Japan). All flow cytometry assays were performed on Beckman Coulter CytoFLEX.

### Chemical Synthesis

Synthesis procedures for photosensitizers and design strategies are shown in Scheme S1.

### Preparation of M@P nanoplatform

4 mg of MTCN-3 was dissolved in 0.5 mL of DMSO, and 1 mg of Poly(I:C) was dissolved in 1 mL of deionized water. The two solutions were mixed and sonicated for three minutes (40 W, 20 KHz). 15 mg DSPE-Hyd-PEG-Folate was dissolved in 4.5 mL of DMSO and sonicated until completely dissolved. Then, 100 mL of deionized water was added to the beaker and stirred at 1500 r min^-1^. The mixed solution was added with DSPE-Hyd-PEG-Folate solution and stirred overnight.

### DLS

The hydrodynamic size distribution of the M@P was detected by NanoBrook 90Plus (Brookhaven) at 25 °C in aqueous solution and PBS buffer. The concentration of M@P was 0.2 mg mL^-1^.

### Zeta potential

NanoBrook 90Plus (Brookhaven) was used to detect the zeta potential of M@P. All the results were measured in aqueous solution at 25 °C. The concentration of M@P was 0.2 mg mL^-1^.

### Cell culture

MDA-MB-231 cells, 4T1 cells, and RAW 264.7 cells were cultured in medium containing DMEM, 10% FBS and antibiotics (100 units mL^-1^ penicillin and 100 μg mL^-1^ streptomycin) in a 5% CO_2_ humility incubator at 37°C.

### Animal model

4T1 mouse model was purchased from the animal center of Jiangsu Keygen Biotechnology Co., Ltd. and operated according to the guidelines provided by the Institutional Animal Care and Use Committee (IACUC). The approval or accreditation number is IACUC-202402111. 4T1 cells (1 × 10^6^) injected into each mouse to establish 4T1 tumor model. In vivo experiments were performed when the tumor size was about 100 mm^3^. For the combined therapy of phototherapy, photothermal, and photoimmunotherapy, we used Balb/C mice to construct a subcutaneous tumor model of breast cancer (4T1 cells). Unilateral 4T1 tumor-bearing mice with tumors approximately 100 mm^3^ were randomly divided into three groups (PBS, M@P, M@P + L). After injecting M@P (10 mg kg^-1^) for 6 h every day, the tumor site was exposed to light (520 nm, 0.5 W cm^-2^, 5 min), and the mouse body weight and tumor volume were measured every 2 days. After 9 days, the mice in each group were killed, and the tumors were separated and photographed. Bilateral 4T1 tumor-bearing mice with tumors approximately 100 mm^3^ were randomly divided into four groups: PBS, M@P, M + L, and M@P + L. Only the primary tumors on the right side were treated and the growth of the primary and distant tumors were monitored every 2 days. The primary tumors in M + L group and M@P + L group were irradiated (520 nm, 0.5 W cm^-2^, 5 min) after intratumor injection (10 mg kg^-1^) 2 h. After 9 days, the mice in each group were killed, and the tumors were separated and photographed.

## Supplementary information

Supplementary Information

## Data Availability

All data used in this study are available from the corresponding authors upon reasonable request.
