# Supplementary material for: A self-assembling nanoplatform for pyroptosis and ferroptosis enhanced cancer photoimmunotherapy
Source: Light Sci Appl. 2025 Jan 2;14:16. doi: 10.1038/s41377-024-01673-1 (PMC11693763; doi:10.1038/s41377-024-01673-1)
Supplement: Supplementary file 1 — Supplementary Information [file 41377_2024_1673_MOESM1_ESM.pdf]

Supplementary Information

A Self-assembling Nanoplatform for Pyroptosis and Ferroptosis Enhanced Cancer Photoimmunotherapy

Zhichao Wang[1], Yuqi Tang[1], and Quan Li[1,2]**

[1]Institute of Advanced Materials and School of Chemistry and Chemical Engineering, Southeast University, Nanjing 211189, China

*E-mail: Yuqi Tang (yqtang@seu.edu.cn) or Quan Li (quanli3273@gmail.com)

[2]Materials Science Graduate Program, Kent State University, Kent, OH 44242, USA

Supplementary Chemical Synthesis

Synthesis of compound b[1]

(3-Bromopropyl) trimethylammonium bromide (a) (2.61 g, 10 mmol) and 4-methylpyridine (0.93 g, 10 mmol) were dissolved into anhydrous N, N-dimethylformamide (DMF, 20 mL) in a 50 mL round flask. The mixture was refluxed for 4 h at 80 °C. After cooling, wash with ether and filter the precipitate to obtain the white solid compound b (2.78 g, 86%). ^{1}H NMR (600 MHz, D_2O) δ 8.87 (d, *J* = 6.6 Hz, 2H), 7.90 (d, *J* = 5.9 Hz, 2H), 4.66-4.62 (m, 2H), 3.37 (s, 11H), 2.61 (s, 3H), 2.53-2.50 (m, 2H).

Synthesis of compound c[1]

4-Bromo-4', 4''-dimethyltriphenylamine (3.38 g, 9.6 mmol), 5-formyl-2-thiopheneboronic acid (1 g, 6.4 mmol), potassium carbonate (35.3 g, 25.6 mmol), and $Pd(PPh_3)_4$ (0.37 g, 0.32 mmol) were dissolved in toluene (Tol, 90 mL)/ methanol (MeOH, 90 mL) mixed solution and removed all air. Then all reaction mixture was stirred at 95 °C for 16 h. After cooling the reaction mixture to room temperature, it was extracted with dichloromethane (DCM) and water and dried with anhydrous Na_2SO_4. After evaporation of the solvent, the crude product was purified by silica gel column chromatography using petroleum ether (PE)/ethyl acetate (EA) (10/1, v/v) as eluent to obtain a yellow solid (1.53 g, 62.3%). ^{1}H NMR (600 MHz, $CDCl_3$) δ 9.84 (s, 1H), 7.69 (d, *J* = 3.8 Hz, 1H), 7.47 (d, *J* = 8.6 Hz, 2H), 7.27 (d, *J* = 3.9 Hz, 1H), 7.10 (d, *J* = 8.0 Hz, 4H), 7.03 (d, *J* = 8.1 Hz, 4H), 6.99 (d, *J* = 8.6 Hz, 2H), 2.33 (s, 6H).

Synthesis of compound d

(3-Bromopropyl) trimethylammonium bromide (a) (2.61 g, 10 mmol) and 3-pyridineacetonitrile (1.18 g, 10 mmol) were dissolved into anhydrous acetonitrile (ACN, 20 mL) in a 50 mL round flask. The mixture was refluxed for 4 h at 80 °C. After cooling to room temperature, the solvent in the reaction system was removed by rotary evaporation, washed with ether, and filter the precipitate to obtain the white solid compound d (2.80 g, 74%). ^{1}H NMR (600 MHz, DMSO-d_6) δ 9.35 (s, 1H), 9.25 (d, *J* = 6.1 Hz, 1H), 8.66 (d, *J* = 8.1 Hz, 1H), 8.33-8.20 (m, 1H), 4.75 (t, *J* = 7.5 Hz, 2H), 4.49 (s, 2H), 3.14 (s, 9H), 3.12 (s, 2H), 2.53 (m, 2H). ^{13}C NMR (151 MHz, DMSO-*d6*) δ 145.93, 144.84, 133.16, 128.60, 117.80, 62.05, 58.30, 52.91, 24.67, 20.92.

Synthesis of monomer MTCN-1

Compound c (0.38 g, 1 mmol) and 3-pyridineacetonitrile (0.12 g, 1 mmol) were added to anhydrous ethanol (EtOH) (30 mL), piperidine (4.26 mg, 0.05 mmol) was added as a catalyst and refluxed (78 °C) for 4 h. It was then cooled to room temperature and the solvent was removed by evaporation under reduced pressure. The yellow target product MTCN-1 could be obtained by continuously washing the residue with EtOH, with a yield of 61.23%. ^{1}H NMR (600 MHz, DMSO-d_6) δ 8.92 (d, *J* = 1.9 Hz, 1H), 8.58 (d, *J* = 4.7 Hz, 1H), 8.36 (s, 1H), 8.08 (d, *J* = 7.9 Hz, 1H), 7.74 (d, *J* = 3.9 Hz, 1H), 7.61 (d, *J* = 8.6 Hz, 2H),

7.55-7.51 (m, 2H), 7.16 (d, J = 8.1 Hz, 4H), 6.99 (d, J = 8.2 Hz, 4H), 6.91 (d, J = 8.6 Hz, 2H), 2.29 (s, 7H). ^{13}C NMR (151 MHz, DMSO-d_6) δ 149.79, 149.72, 148.94, 146.81, 144.50, 137.80, 137.50, 135.67, 133.72, 133.17, 130.70, 130.21, 127.43, 125.56, 124.42, 123.64, 121.30, 118.13, 102.03, 20.92. HR-ESI-MS: calcd. for $C_{32}H_{25}N_3S$: m/z: 484.1803, found: m/z 484.18096.

Synthesis of monomer MTCN-2

Compound b (0.35 g, 1 mmol) and compound c (0.38 g, 1 mmol) were added to anhydrous EtOH (30 mL), piperidine (4.26 mg, 0.05 mmol) was added as a catalyst, and refluxed (78 °C) for 7 h in N_2 atmosphere. It was then cooled to room temperature and the solvent was removed by evaporation under reduced pressure. Using DCM/MeOH (10/1, v/v) as eluent, the crude product was purified by silica gel column chromatography, and the purple solid product was obtained with a yield of 55.12%. ^{1}H NMR (600 MHz, DMSO-d_6) δ 8.93 (s, 2H), 8.29-8.21 (m, 3H), 7.58 (d, J = 8.6 Hz, 2H), 7.53-7.47 (m, 2H), 7.18-7.13 (m, 5H), 6.99 (d, J = 8.1 Hz, 4H), 6.90 (d, J = 8.3 Hz, 2H), 4.54 (t, J = 7.0 Hz, 2H), 3.09 (s, 11H), 2.45-2.40 (m, 2H), 2.29 (s, 6H). ^{13}C NMR (151 MHz, DMSO-d_6) δ 153.52, 151.81, 148.77, 148.24, 144.48, 138.86, 134.91, 134.55, 133.75, 130.72, 127.20, 125.59, 124.39, 123.69, 121.43, 121.20, 62.26, 56.86, 52.96, 24.58, 20.93. HR-ESI-MS: calcd. for $C_{37}H_{41}Br_2N_3S$: m/z: 1/2[M-2Br]$^{2+}$: 279.6505, found: m/z 279.64970.

Synthesis of monomer MTCN-3

Compound b (0.35 g, 1 mmol) and compound d (0.40 g, 1 mmol) were added to anhydrous EtOH (30 mL), piperidine (4.26 mg, 0.05 mmol) was added as a catalyst, and refluxed (78 °C) for 7 h in N_2 atmosphere. It was then cooled to room temperature and the solvent was removed by evaporation under reduced pressure. Using DCM/MeOH (2/1, v/v) as eluent, the crude product was purified by silica gel column chromatography, and the dark red solid product was obtained with a yield of 50.23%. ^{1}H NMR (600 MHz, DMSO-d_6) δ 9.66 (s, 1H), 9.13 (d, J = 5.9 Hz, 1H), 8.89 (s, 1H), 8.83 (d, J = 8.4 Hz, 1H), 8.29 (dd, J = 8.0, 6.2 Hz, 1H), 7.93 (d, J = 4.0 Hz, 1H), 7.65 (t, J = 6.1 Hz, 3H), 7.18 (d, J = 8.2 Hz, 4H), 7.01 (d, J = 8.2 Hz, 4H), 6.92 (d, J = 8.7 Hz, 2H), 4.79 (dd, J = 13.2, 6.0 Hz, 2H), 3.48 (dd, J = 10.6, 5.5 Hz, 2H), 3.13 (s, 9H), 2.57 (dt, J = 15.3, 7.7 Hz, 2H), 2.29 (s, 6H). ^{13}C NMR (151 MHz, DMSO) δ 152.27, 149.39, 144.32, 143.79, 142.37, 141.58, 141.54, 140.16, 135.13, 134.82, 133.98, 130.75, 128.65, 127.72, 125.73, 124.97, 124.10, 120.93, 117.35, 98.08, 62.25, 58.59, 53.06, 24.87, 20.93. HR-ESI-MS: calcd. for $C_{38}H_{40}Br_2N_4S$: m/z: 1/2[M-2Br]$^{2+}$: 292.1482, found: m/z 292.14551.

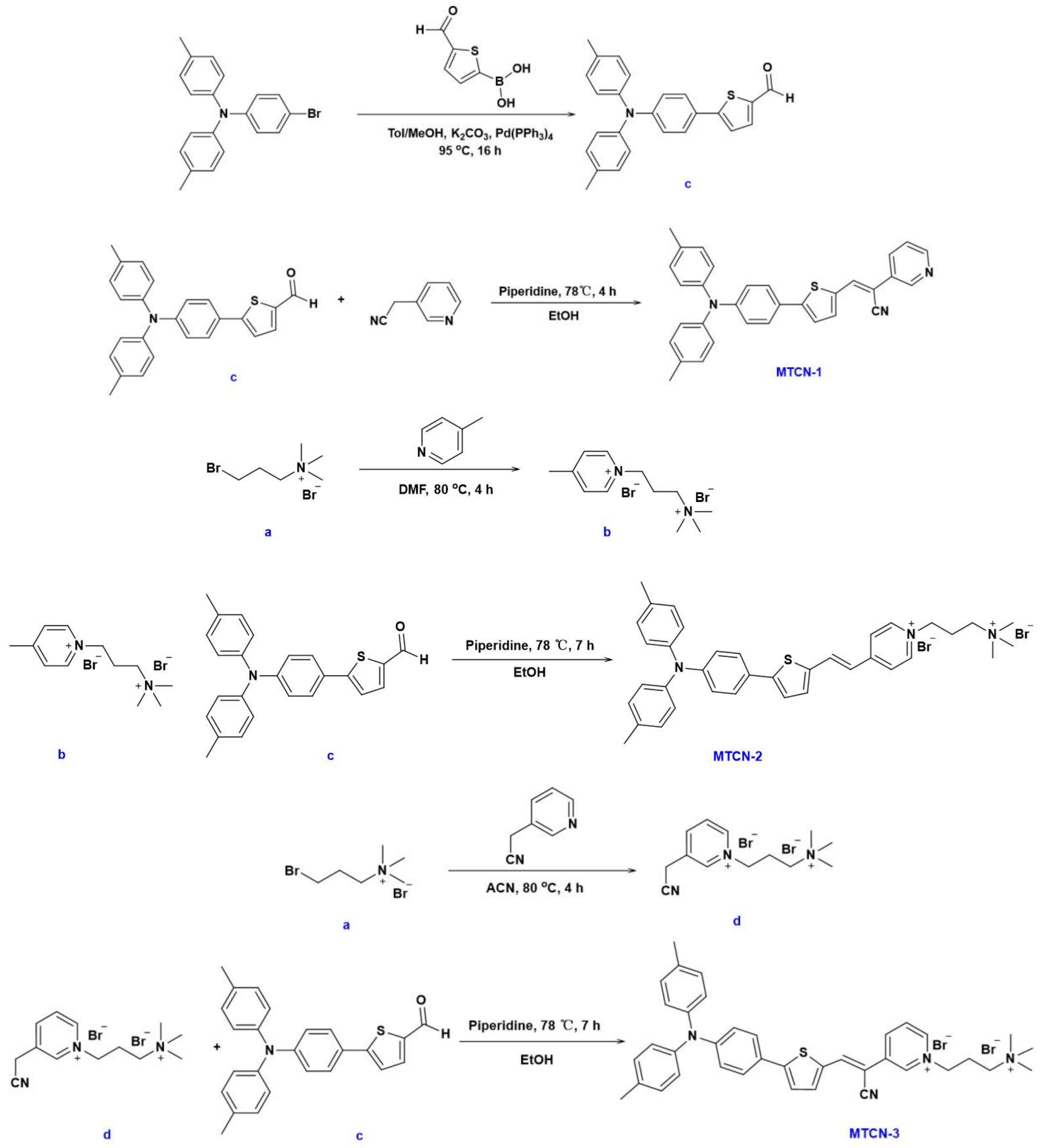

Scheme S1. The synthesis routes of target compounds **MTCN-1**, **MTCN-2**, and **MTCN-3**.

Supplementary Experimental methods

Reactive oxygen species (ROS) detection

The production of ROS was studied with 9,10-anthracenyl-bis (methylene) imalonic acid (ABDA) as $^1O_2$ indicator, hydroxyphenyl fluorescein (HPF) as •OH indicator, dihydrorhodamine 123 (DHR 123) as $O_2^{\bullet-}$ indicator and 2’,7’-dichlorodihydrofluorescein (DCFH) as ROS indicator. A mixed solution containing ABDA and Aggregation-induced emission luminogens (AIEgens) was exposed to white light (10 mW cm^{-2}). Record the absorbance of ABDA at 378 nm on the UV-Vis spectrophotometer at different

irradiation times. The final concentration of AIEgens and HPF, DHR 123, or DCFH in aqueous solution were both 10 μM. Then it was exposed to white light (10 mW cm^{-2}) for different times, and the emission spectrum was observed.

Photothermal performance measurement

An infrared thermal imager was used to detect the photothermal effect of AIEgens. The temperature was then naturally cooled to room temperature, and temperature curves were recorded every 10 s.

The photothermal conversion efficiency was calculated as follows:

$$\eta = \frac{hS(T_{Max} - T_{Sur}) - Q_{Dis}}{I(1 - 10^{-A_\lambda})}$$

T_{Max} and T_{Sur}, are the maximum equilibrium temperature and ambient temperature, respectively. Q_{Dis} represents the heat emitted by the light absorbed by the sample cell. I is the laser power. To determine the photothermal conversion efficiency (η), it is also necessary to obtain the heat transfer efficiency (h) and the surface area of the vessel (S). To derive (*hS*), dimensionless driving temperature (θ) and time constant (τ), formulas are introduced. m_d and C_d are the mass and specific heat capacity of each component in solution.

$$\theta = \frac{T - T_{Sur}}{T_{Max} - T_{Sur}}$$

$$t = -\tau \ln(\theta)$$

$$\tau = \frac{m_d C_d}{hS}$$

Cellular uptake and intracellular ROS detection

MDA-MB-231 cells were incubated with M@P in confocal dish for 5 h. Then the solution of the M@P was discarded and the cells were washed with phosphate buffered saline PBS (3 mL). These samples were further incubated with 2,7-dichlorodihydrofluorescein diacetate (DCFH-DA, 10 μM) for another 10 min and washed with PBS for three times (1 mL). They were irradiated for 5 min. As the control group, cells were incubated with PBS in confocal dishes. Fluorescence images were recorded on a confocal laser scanning microscope (CLSM). Excitation wavelength: 488 nm.

Live/dead cell staining

MDA-MB-231 cells were treated with M@P for 5 h. After that, the fresh medium was used to replace the medium, and then the cells were irradiated with 520 nm laser (0.5 W cm^{-2}) for 5 min, then stained with propidium iodide (PI) and calcein-AM for 10 min, washed with PBS for three times, and fluorescence images were recorded on CLSM imaging.

MTT assay

To evaluate the biosafety, the cytotoxicity was assessed through MTT assay. Cells were seeded in 96-well plates at a density of 5×10^4 cells per well and cultured in standard medium for 24 h. The cells were then incubated with various concentrations (5, 10, 20, 40, 60, 80 mg mL^{-1}) in the dark for 24 h. After cells were washed by PBS twice, 100 μL of freshly prepared MTT solution (1 mg mL^{-1}) was added into each well. The MTT solution was carefully removed after 3 h of incubation, and 150 μL of DMSO was added into each well to dissolve all the purple crystals formed. The absorbance of MTT at 570 nm was measured by the microplate reader (VarioskanTM LUX multi-mode microplate reader). Each of above experiment was repeated three times.

Acridine orange (AO) staining

MDA-MB-231 cells were seeded into confocal dishes and cultured for 24 h, and then incubated with PBS (control), M@P, M@P+L (520 nm, 0.5 W cm^{-2}, 5 min) for 24 h. Next, those cells were stained with AO (2 μM) for 15 min. After washing three times with PBS, the intracellular fluorescent emission of AO was observed by CLSM.

Intracellular pH changes

MDA-MB-231 cells were seeded into confocal dishes and cultured for 24 h, and then incubated with PBS (control), M@P, M@P+L (520 nm, 0.5 W cm^{-2}, 5 min) for 24 h. Next, those cells were stained with BCECF-AM (2.5 μM) for 30 min. After washing three times with PBS, the intracellular fluorescent emission of BCECF-AM was observed by CLSM.

Immunofluorescence

MDA-MB-231 cells were seeded into confocal dishes and cultured for 24 h, and then incubated with PBS (control), M@P, M@P+L (520 nm, 0.5 W cm^{-2}, 5 min) for 24 h, fixed with 4% paraformaldehyde and permeabilized with 0.1% Triton X-100. Then, caspase-1 polyclonal antibody, anti-CRT, HMGB-1 antibodies, GPX4 antibodies, Cathepsin B antibodies, and fluorescein isothiocyanate (FITC)-conjugate secondary antibodies were used. Then the cells were counterstained with hoechst 33342 and observed under CLSM.

Enzyme-linked immunosorbent assay (ELISA) test

MDA-MB-231 cells were seeded into confocal dishes and cultured for 24 h, and then incubated with PBS (control), M@P, M@P+L (520 nm, 0.5 W cm^{-2}, 5 min) for 4 h. After that, the cells were lysed and the supernatant was collected by centrifugation. Firstly, prepare standard, sample and sample diluent. Then, add standard and sample diluent to the microplate containing antibodies to the protein to be tested, and incubate for 30 min at 37°C. Thirdly, wash 5 times, add HRP-conjugate reagent, and incubate for 30 min at 37°C. Then, wash 5 times, add chromogen solution A and B, and incubate for 10 min at 37°C. Finally,

add stop solution and read absorbance at 450 nm within 15 min.

Transwell assay

Migration assays were performed using transwell chamber. Briefly, 2×10^4 RAW 264.7 cells were suspended in 200 μL DMEM medium with 10% fetal bovine serum (FBS) and then seeded into the upper chamber. The lower chamber was filled with 700 μL of cell supernatant after treatment with different compounds and light. After 48 h, the RAW 264.7 cells on the upper transwell chamber were removed, and the cells on the lower transwell chamber were fixed with 4% paraformaldehyde, then stained with 0.5% crystal violet.

Supplementary Figures

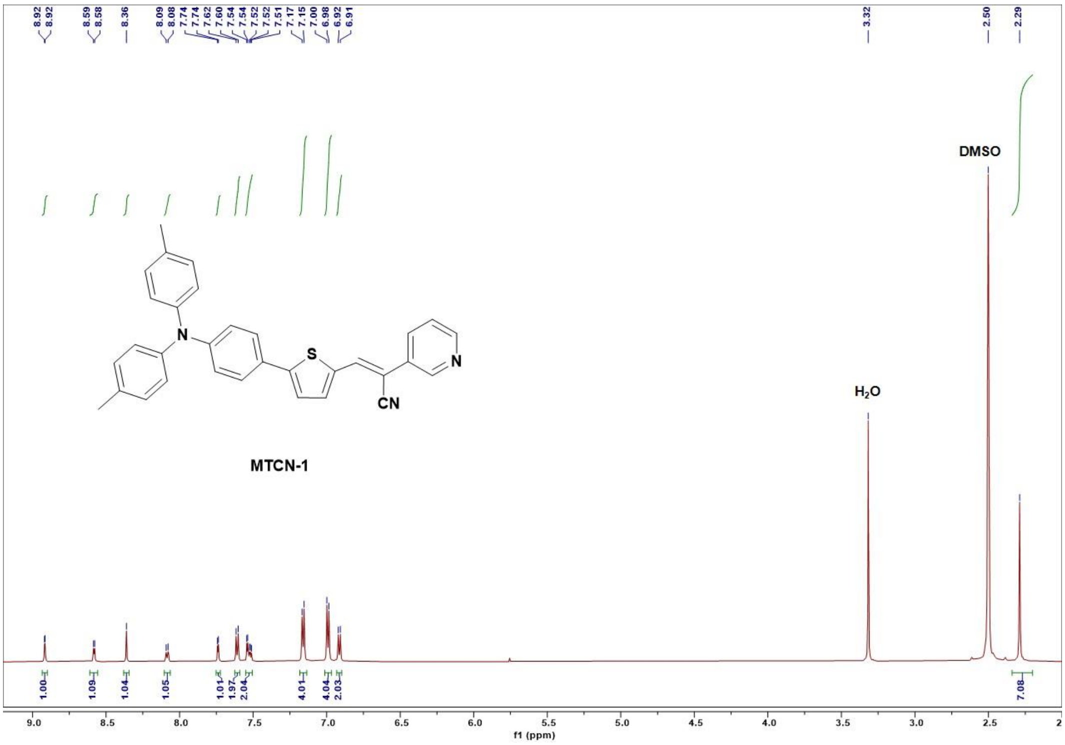

Fig. S1. ¹H NMR (600 MHz, DMSO-*d6*) spectrum of MTCN-1.

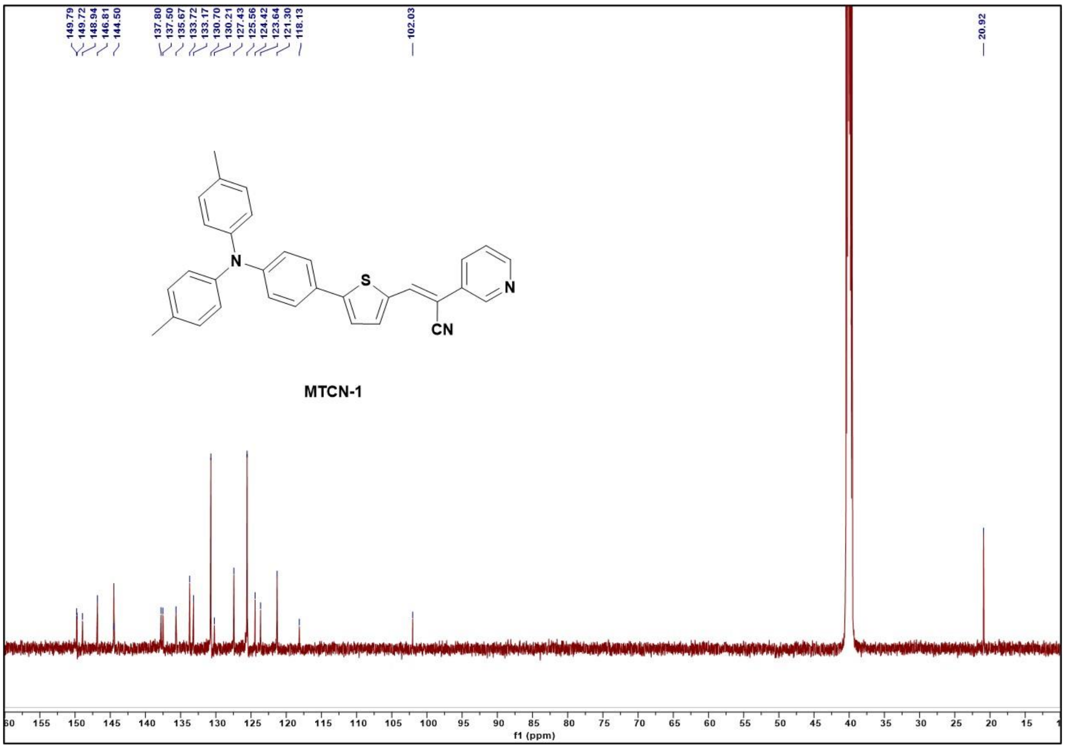

Fig. S2. ¹³C NMR (151 MHz, DMSO-*d6*) spectrum of MTCN-1.

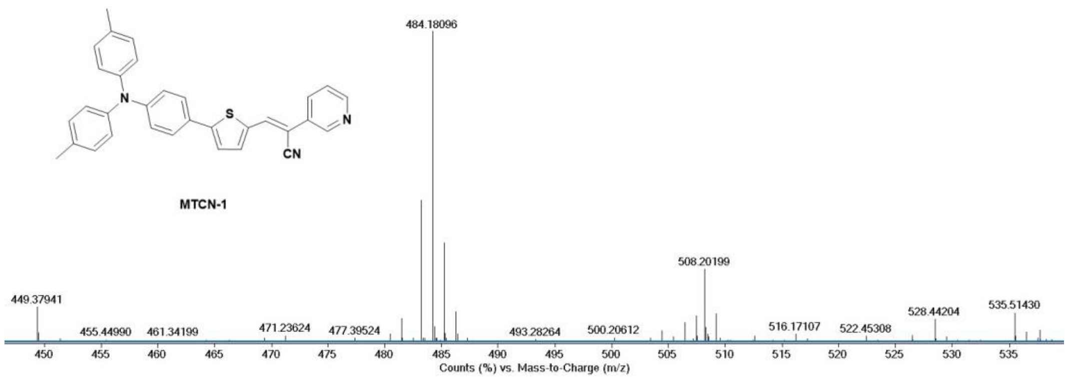

Fig. S3. HRMS spectrum of MTCN-1.

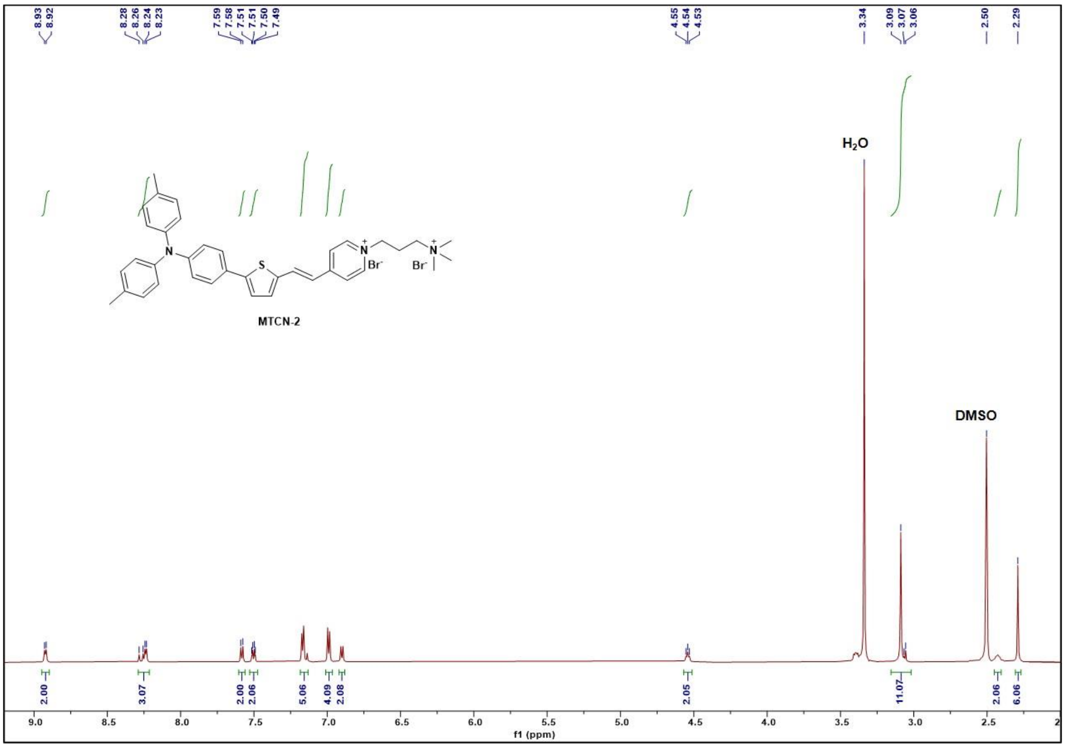

Fig. S4. ^{1}H NMR (600 MHz, DMSO-*d6*) spectrum of MTCN-2.

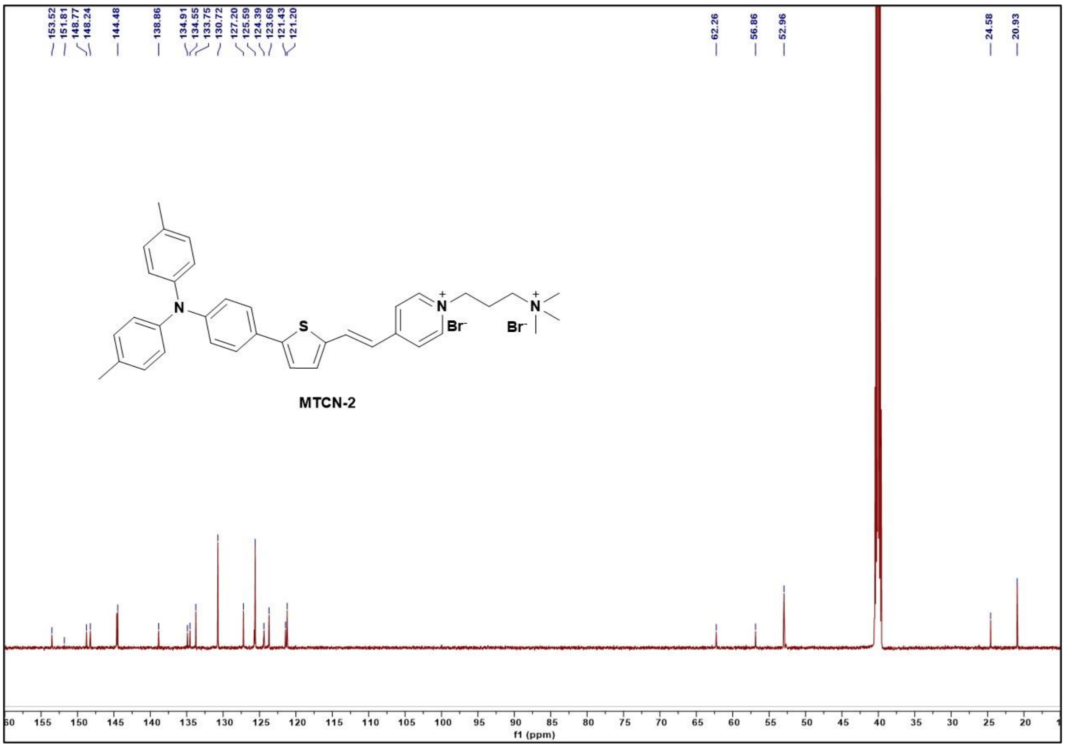

Fig. S5. ^{13}C NMR (151 MHz, DMSO-*d6*) spectrum of MTCN-2.

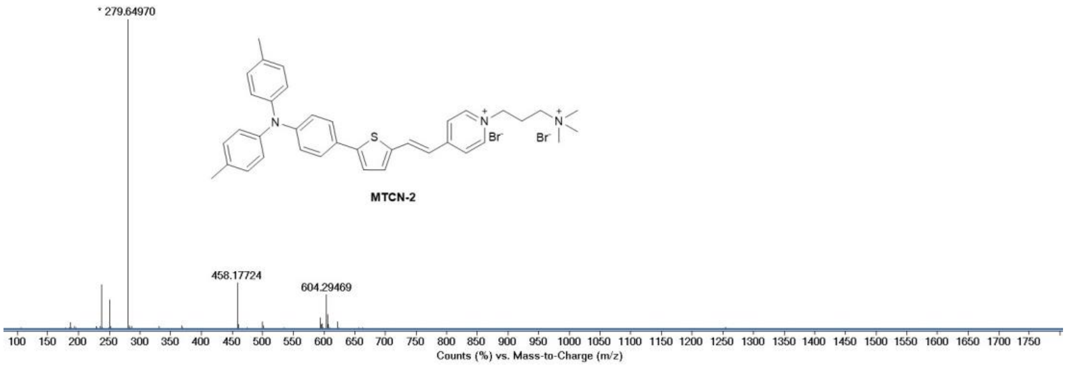

Fig. S6. HRMS spectrum of MTCN-2.

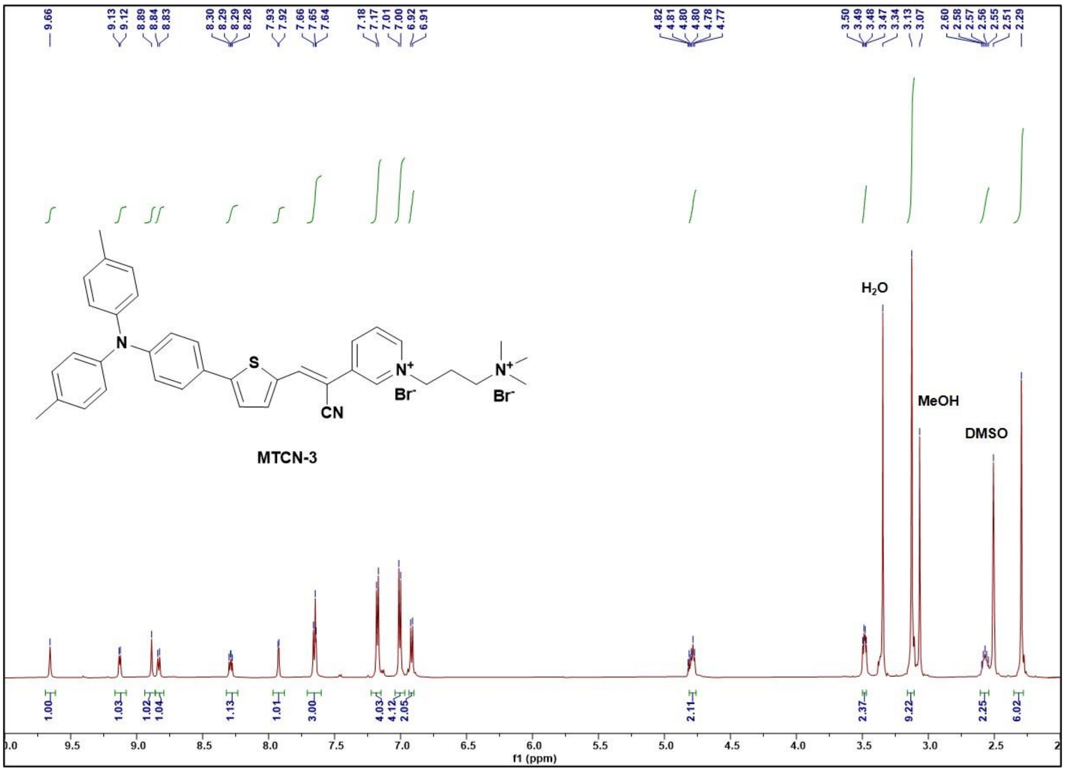

Fig. S7. ^{1}H NMR (600 MHz, DMSO-*d6*) spectrum of MTCN-3.

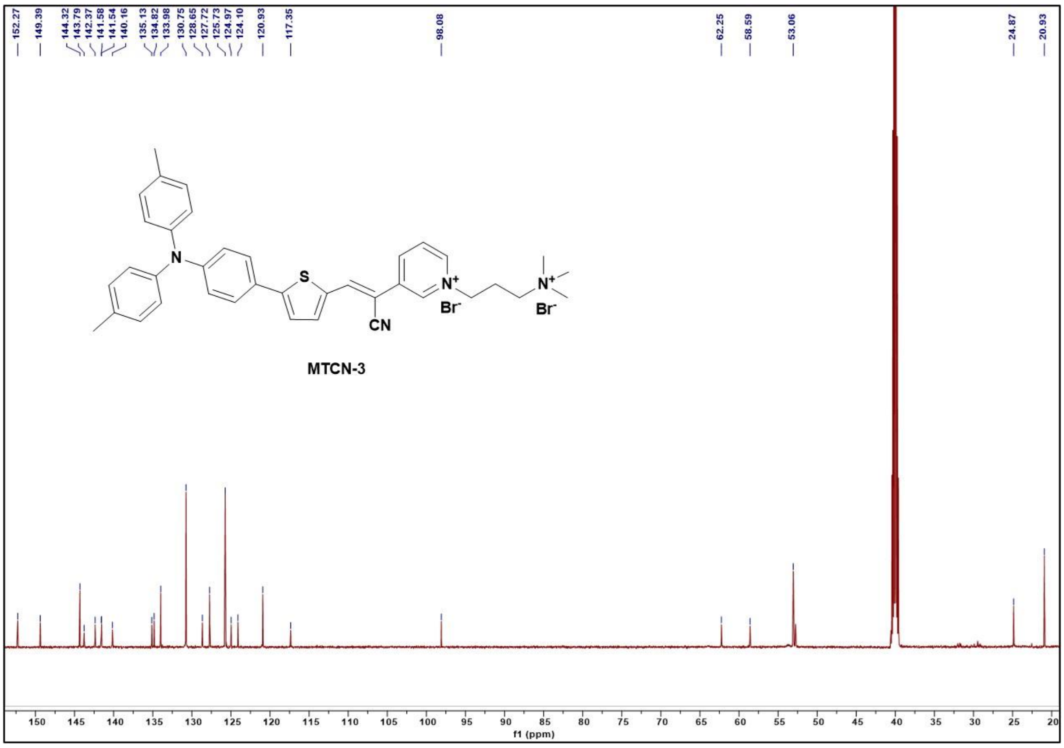

Fig. S8. ^{13}C NMR (151 MHz, DMSO-*d6*) spectrum of MTCN-3.

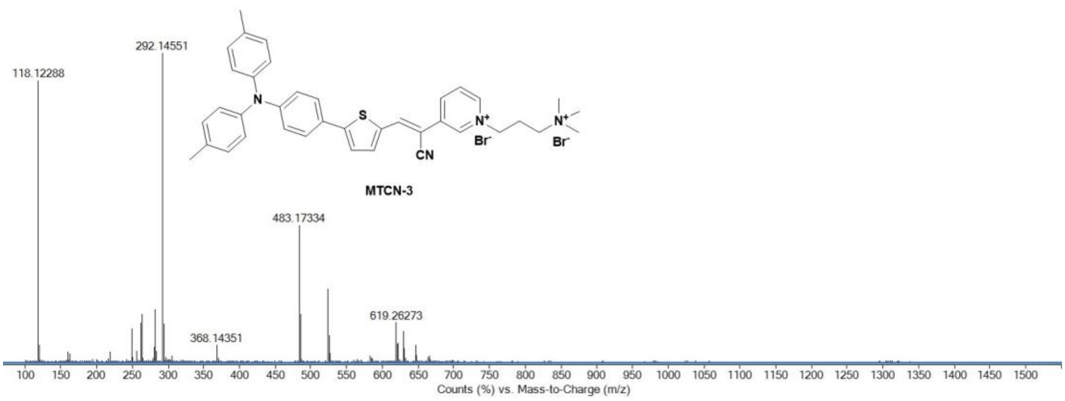

Fig. S9. HRMS spectrum of MTCN-3.

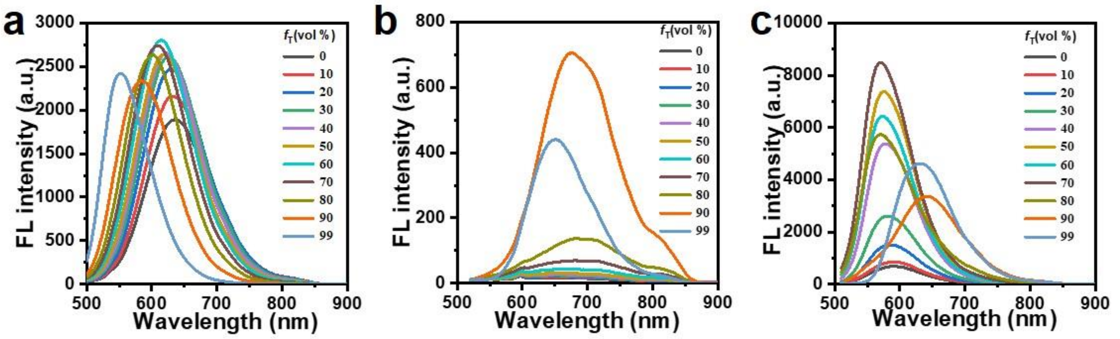

Fig. S10. Fluorescence spectra of a) MTCN-1, b) MTCN-2, and c) MTCN-3 in DMSO/TOL mixtures with different toluene fractions (f_T).

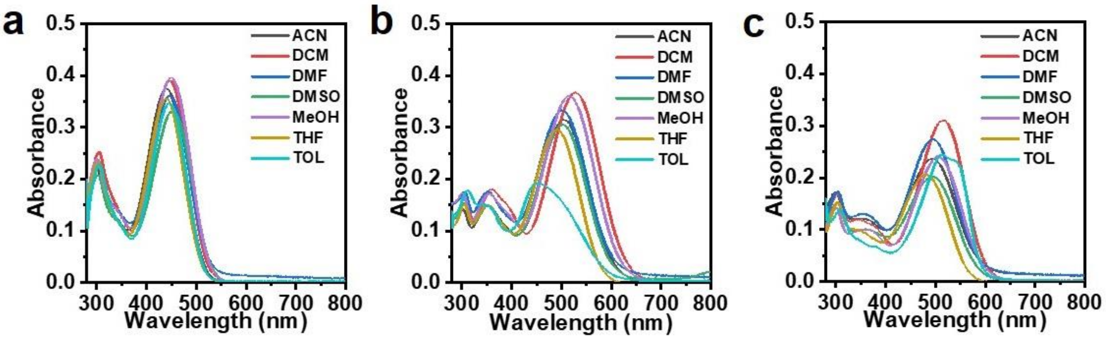

Fig. S11. UV-Vis absorption spectra of a) MTCN-1, b) MTCN-2, and c) MTCN-3 (10 μM) in ACN, DCM, DMF, DMSO, MeOH, tetrahydrofuran (THF), and TOL.

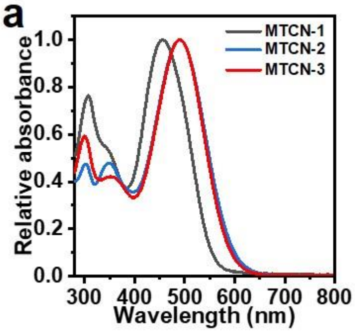

Fig. S12. a) UV-Vis absorption and b) fluorescence emission spectra of MTCN-1, MTCN-2, or MTCN-3 (10 μM) in aqueous solution.

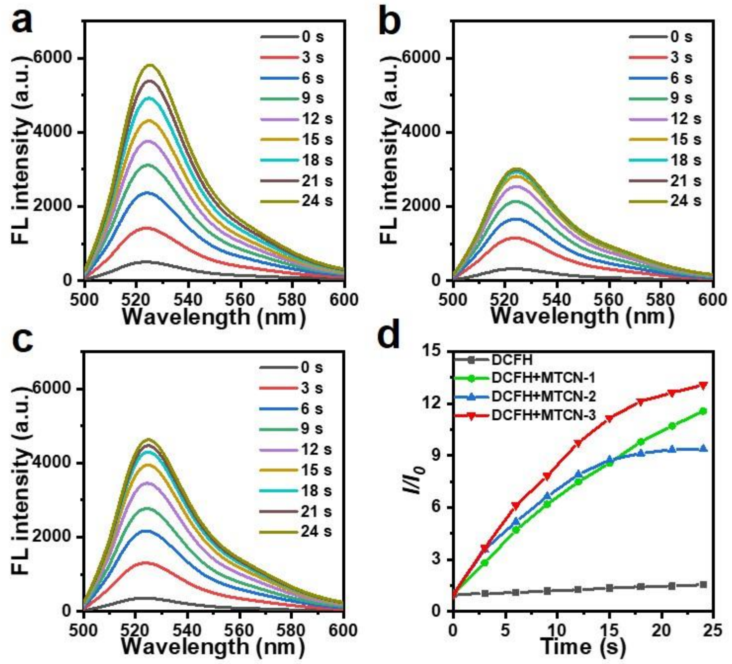

Fig. S13. ROS generation in the absence and presence of a) MTCN-1, b) MTCN-2, and c) MTCN-3 (10 μM), respectively, using DCFH-DA as an indicator; d) Relative change of fluorescence intensity (I/I_0) of DCFH (L: White light, 10 mW cm^{-2}).

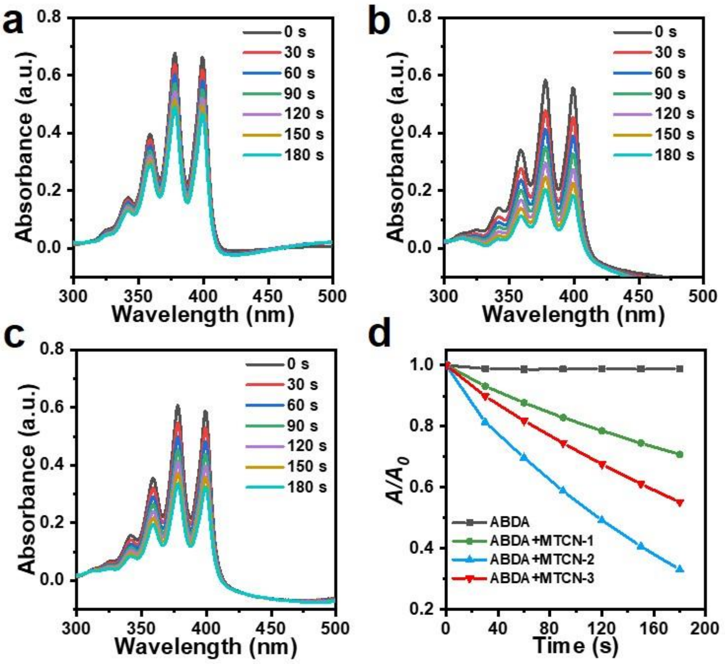

Fig. S14. $^1O_2$ generation in the absence and presence of a) MTCN-1, b) MTCN-2, and c) MTCN-3 (10 μM), respectively, using ABDA as an indicator; d) Relative change of absorbance (A/A_0) of ABDA (L: White light, 10 mW cm^{-2}).

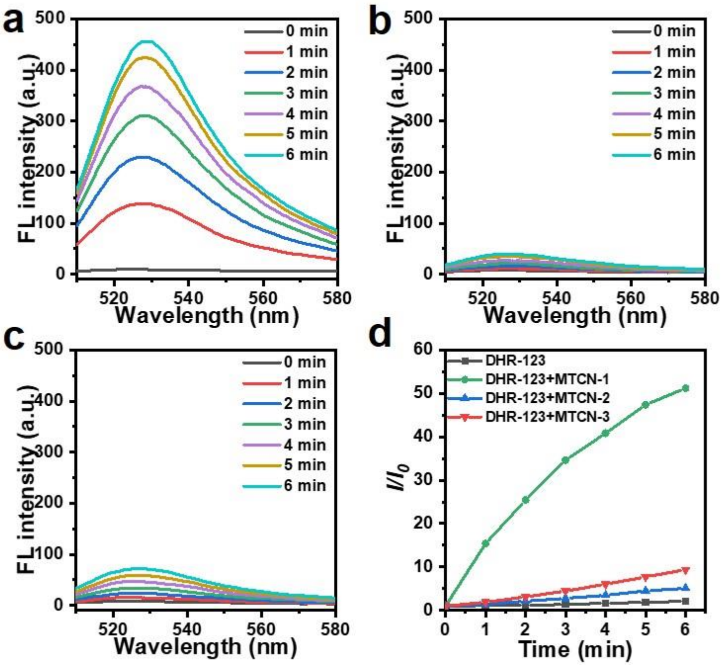

Fig. S15. $O_2^{\bullet -}$ generation in the absence and presence of a) MTCN-1, b) MTCN-2, and c) MTCN-3 (10 μM), respectively, using DHR 123 as an indicator; d) Relative change of fluorescence intensity (I/I_0) of DHR 123 (L: White light, 10 mW cm^{-2}).

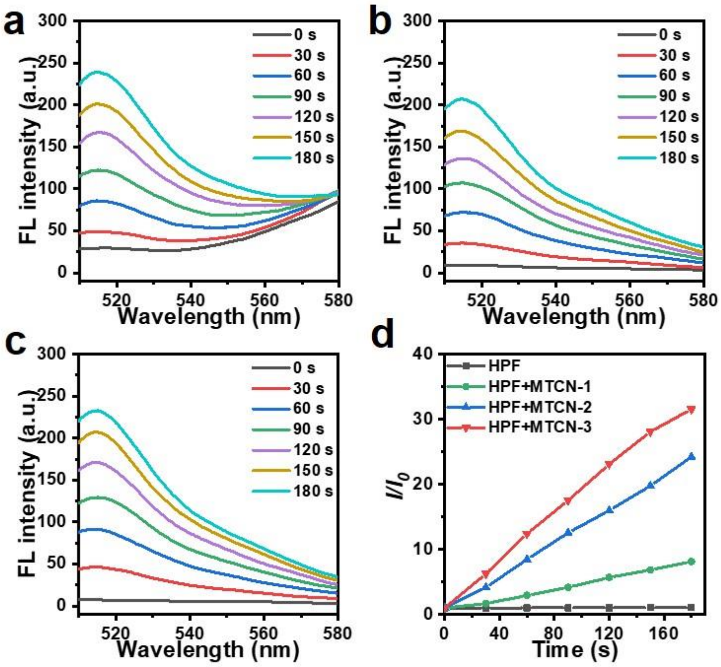

Fig. S16. •OH generation in the absence and presence of a) MTCN-1, b) MTCN-2, and c) MTCN-3 (10 μM), respectively, using HPF as an indicator; d) Relative change of fluorescence intensity (I/I_0) of HPF (L: White light, 10 mW cm^{-2}).

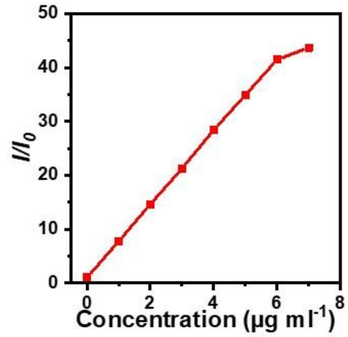

Fig. S17. The relative emission intensity change of MTCN-3 (10 μg mL^{-1}) to Poly(I: C) at different concentrations in aqueous solution.

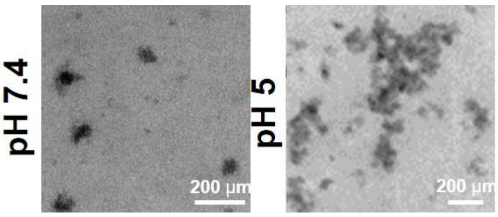

Fig. S18. TEM images of M@P under different pH.

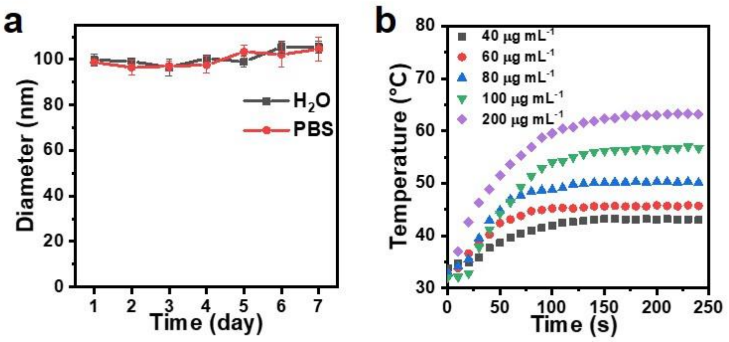

Fig. S19. a) Stability of M@P at different solutions; b) Temperature elevation of M@P at different concentrations under 520 nm laser irradiation (0.5 W cm^{-2}).

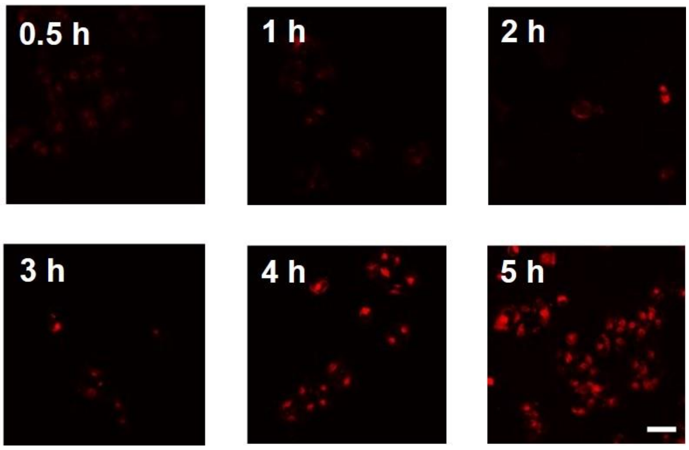

Fig. S20. CLSM images of MDA-MB-231 cancer cells upon incubation with M@P. Scale bar = 50 μm.

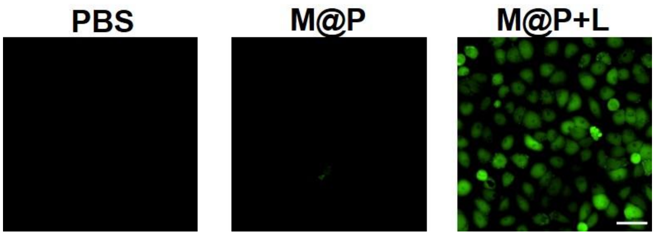

Fig. S21. The M@P-treated MDA-MB-231 cells were irradiated (L: 520 nm, 0.5 W cm^{-2}, 1 min). ROS was detected by DCFH-DA (5 μM). Scare bar = 50 nm.

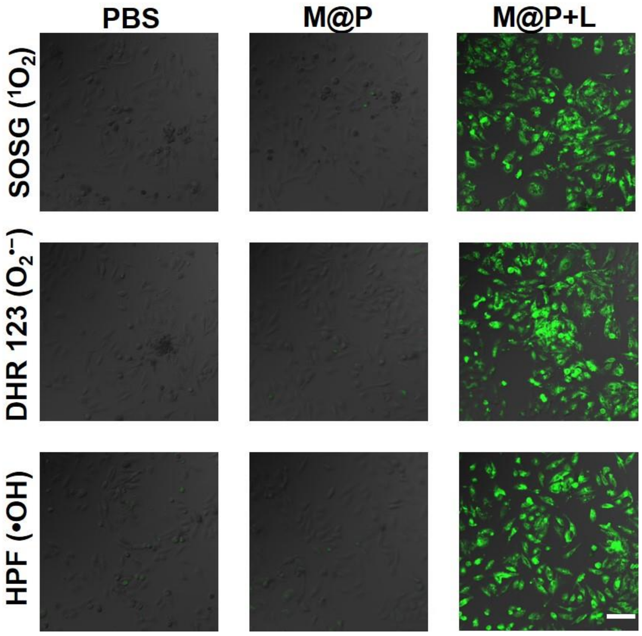

Fig. S22. The M@P-treated MDA-MB-231 cells were irradiated (L: 520 nm, 0.5 W cm^{-2}, 1 min). ROS was detected by SOSG, DHR 123, and HPF (5 μM). Scare bar = 120 nm.

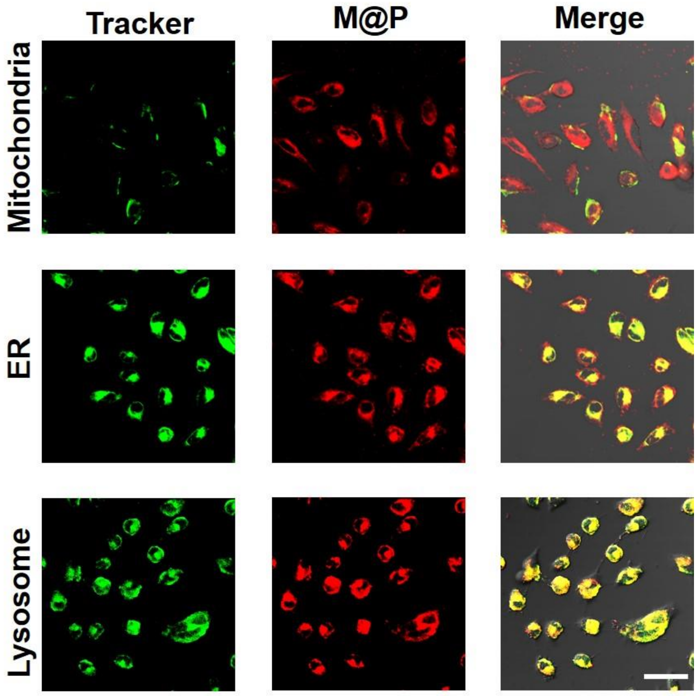

Fig. S23. CLSM images of MDA-MB-231 cells stained with M@P and their co-staining with Mito Tracker Green, ER Tracker Green, and LysoTracker Green (5 μM). Scale bar = 50 μm.

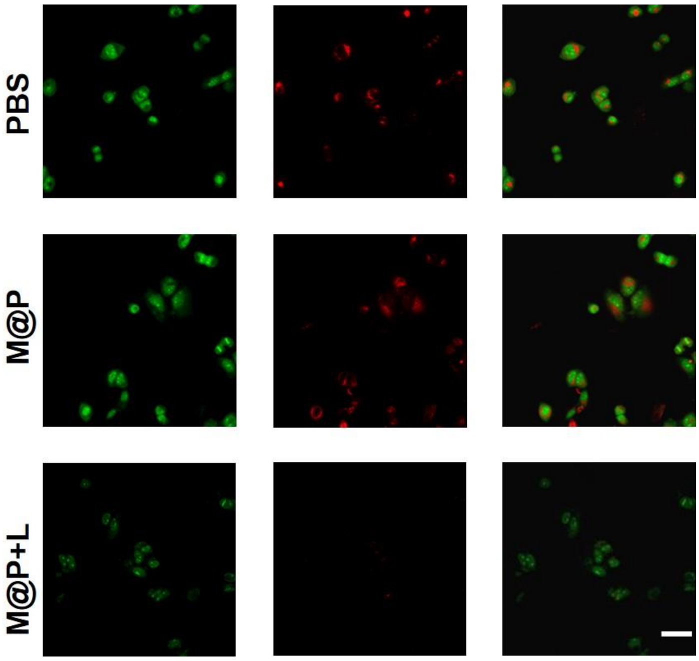

Fig. S24. CLSM images of AO-stained MDA-MB-231 cells treated with M@P (L: 520 nm light, 0.5 W cm^{-2}, 5 min). Scale bar = 50 μm.

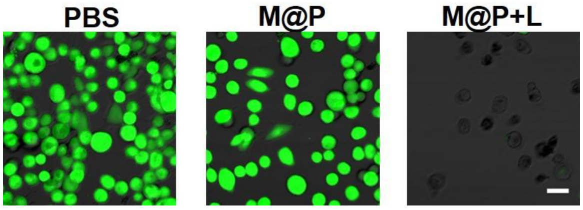

Fig. S25. CLSM images of cancer cells stained by BCECF-AM for detecting intracellular pH. Scale bar = 30 μm.

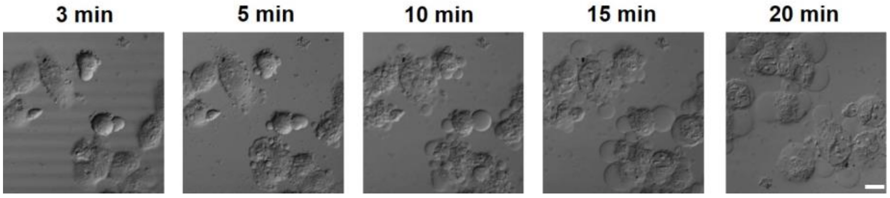

Fig. S26. Real-time observation of M@P-treated MDA-MB-231 cell membrane expansion and content release in pyroptosis. Scale bar = 20 μm.

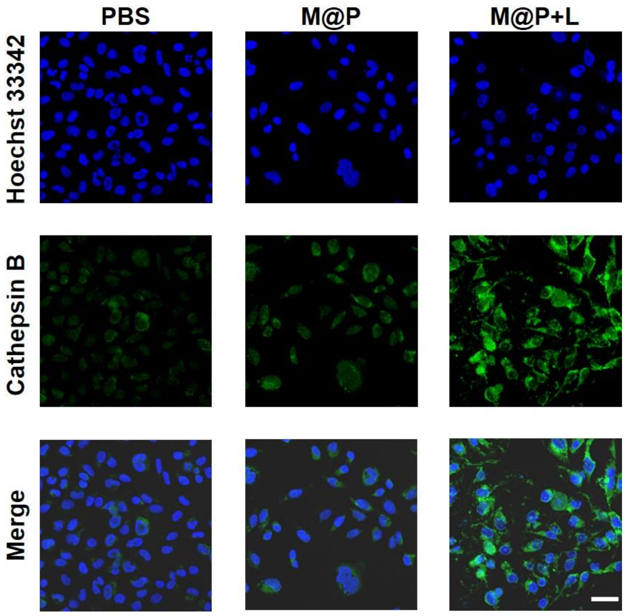

Fig. S27. Immunofluorescent staining of Cathepsin B expression in MDA-MB-231 cells treated with M@P (L: 520 nm light, 0.5 W cm^{-2}, 5 min). Scale bar = 50 nm.

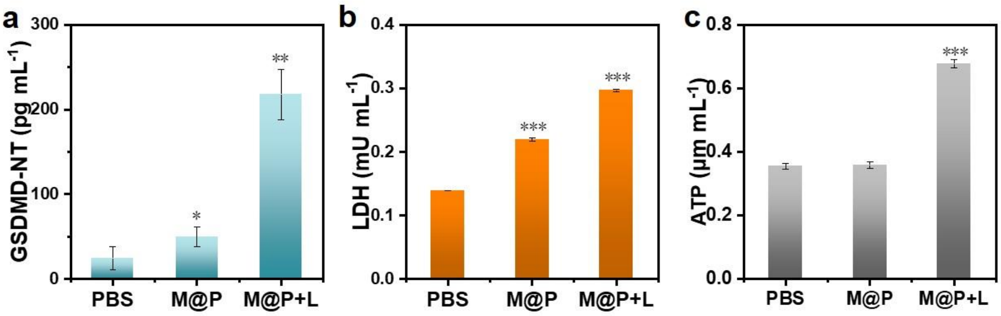

Fig. S28. Quantitative analysis of a) GSDMD-NT, b) LDH, and c) ATP from MDA-MB-231 cells treated with M@P (L: 520 nm light, 0.5 W cm^{-2}, 5 min). Mean ± SD, n = 3, $p > 0.05$, *$p < 0.05$, **$p < 0.01$, ***$p < 0.001$.

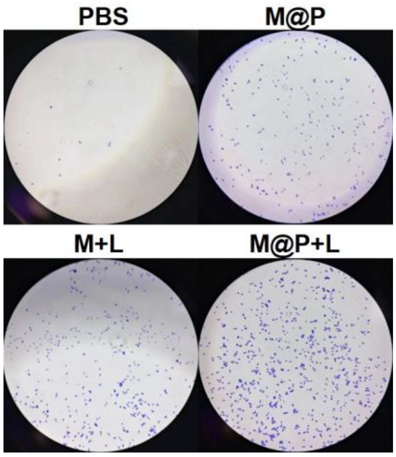

Fig. S29. The supernatant after PDT was co-cultured with RAW 264.7 cells. After co-culture for 24 h, crystalline violet staining was conducted to detect the migration of RAW 264.7 cells.

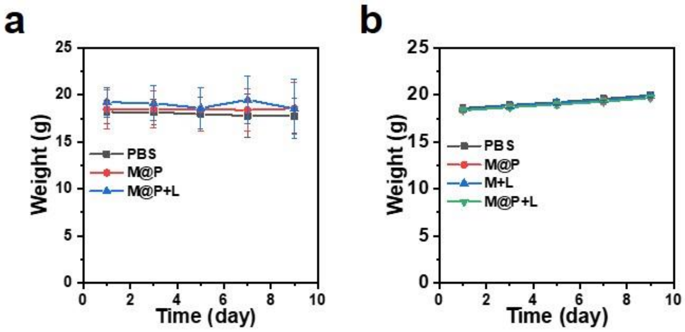

Fig. S30. a) Body-weight changes of 4T1-tumor-bearing mice with different treatments after 9 days; b) Body-weight changes of bilateral 4T1-tumor-bearing mice with different treatments after 9 days.

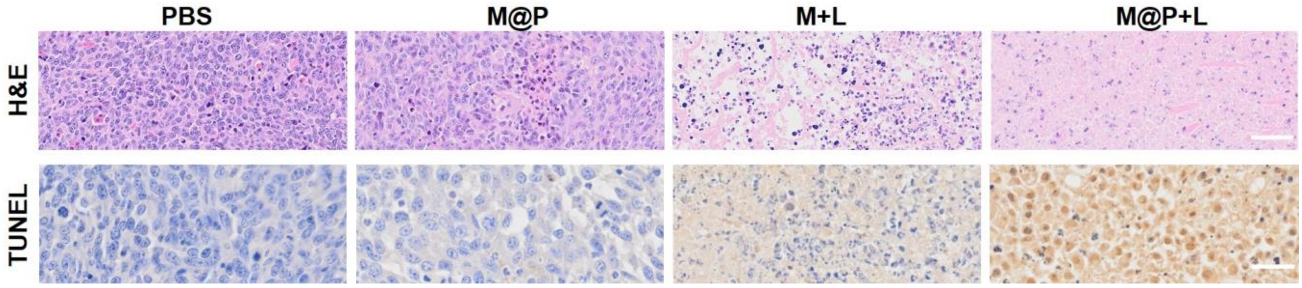

Fig. S31. Hematoxylin-eosin (H&E) staining of the primary tumor from 4T1 tumor-bearing mice after 9 days (Scale bars= 50 μm) and terminal deoxynucleotidyl transferase dUTP nick end labeling (TUNEL) staining of primary tumors from 4T1 tumor-bearing mice after 9 days. (Scale bars = 20 μm)

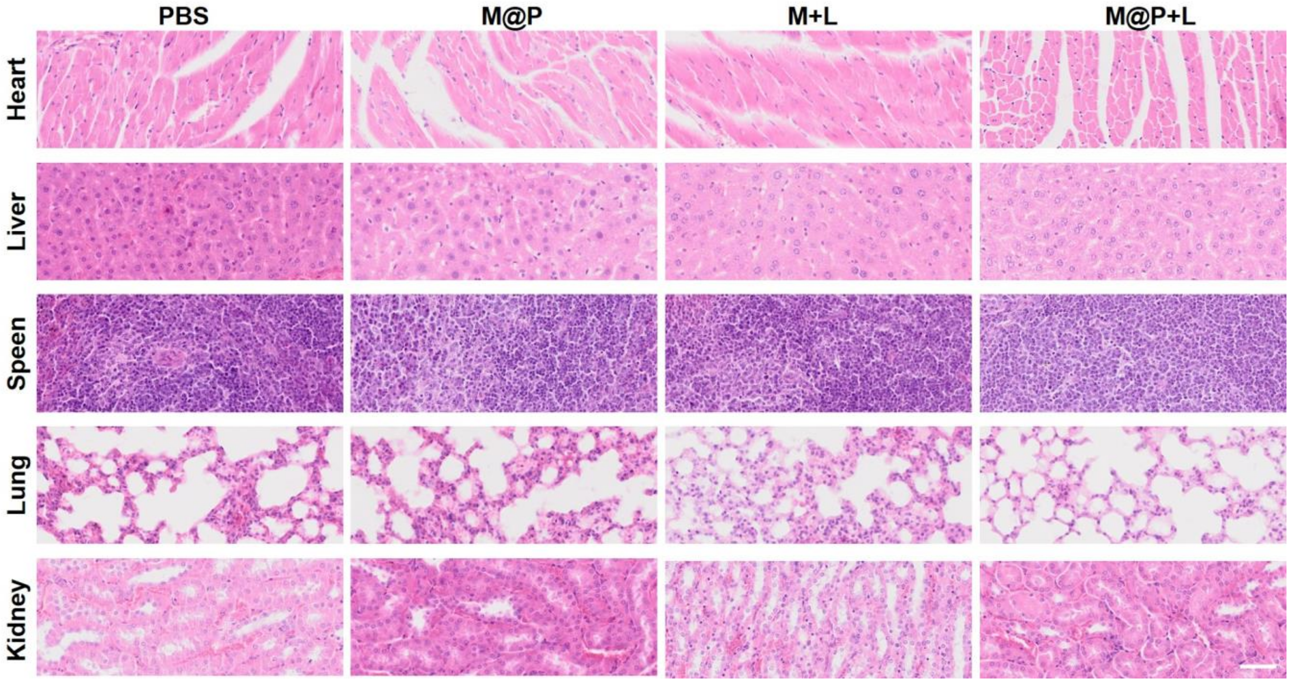

Fig. S32. H&E-stained slice images of major organs of mice after the experiment. Scale bar = 50 nm.

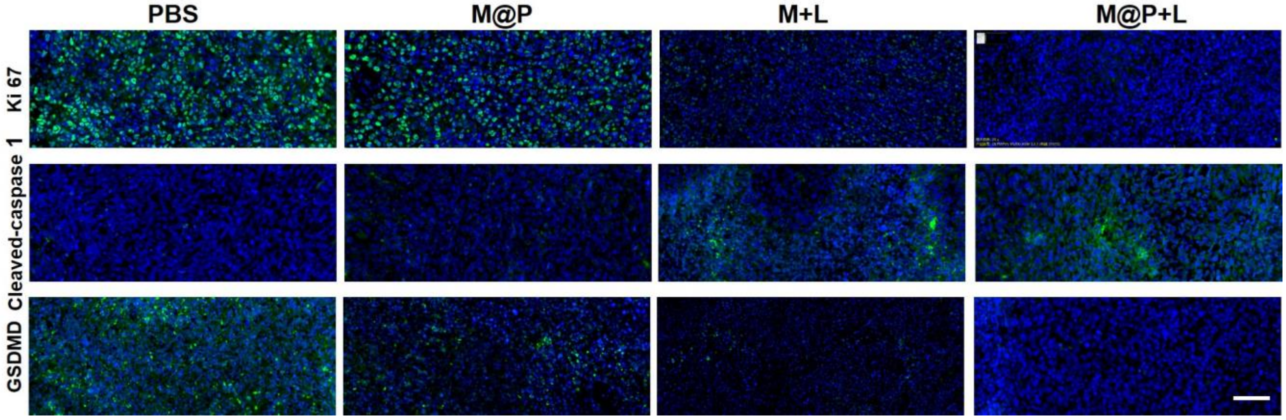

Fig. S33. Ki-67, Cleaved-caspase-1, and gasdermin D (GSDMD) immunostaining images of tumor sections after 9 days of treatment. Scale bar = 50 nm.

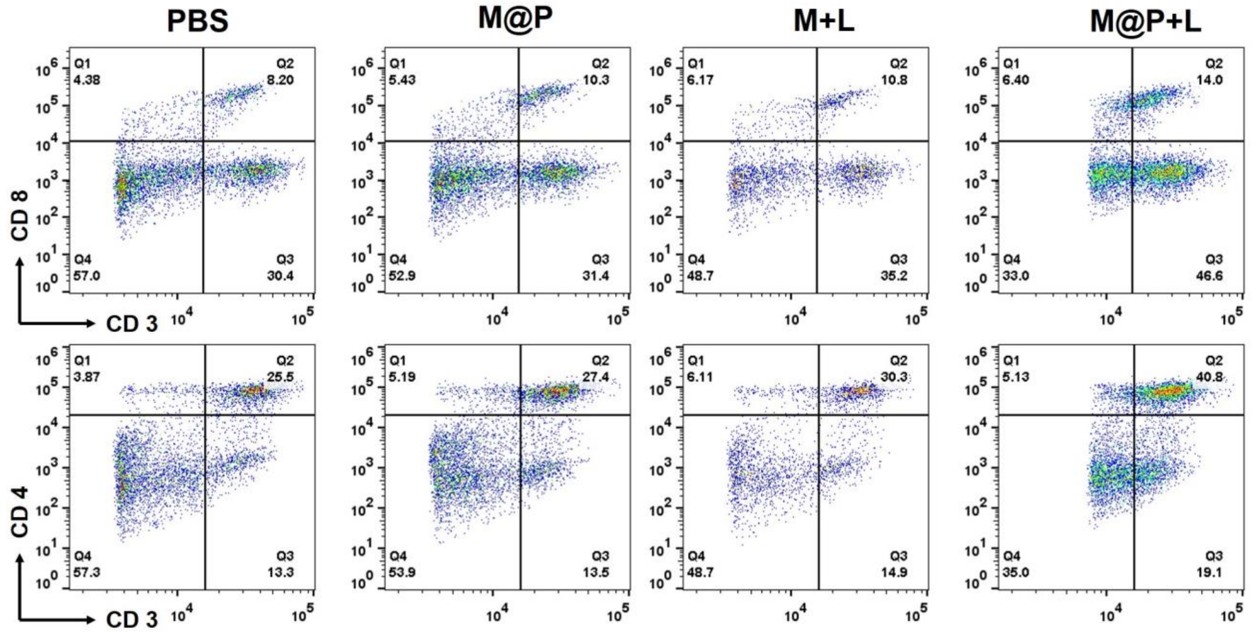

Fig. S34. The proportion of primary tumor infiltrating CD8$^+$ T cells and CD4$^+$ T cells.

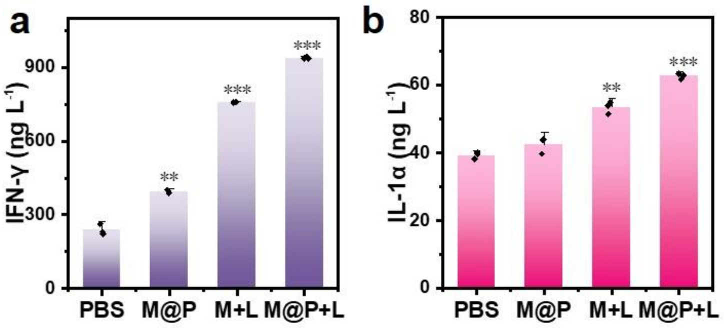

Fig. S35. Quantitative analysis of secretion of a) IFN-γ and b) IL-1α in serum. Mean ± SD, n = 3, *$p < 0.05$, **$p < 0.01$, ***$p < 0.001$.

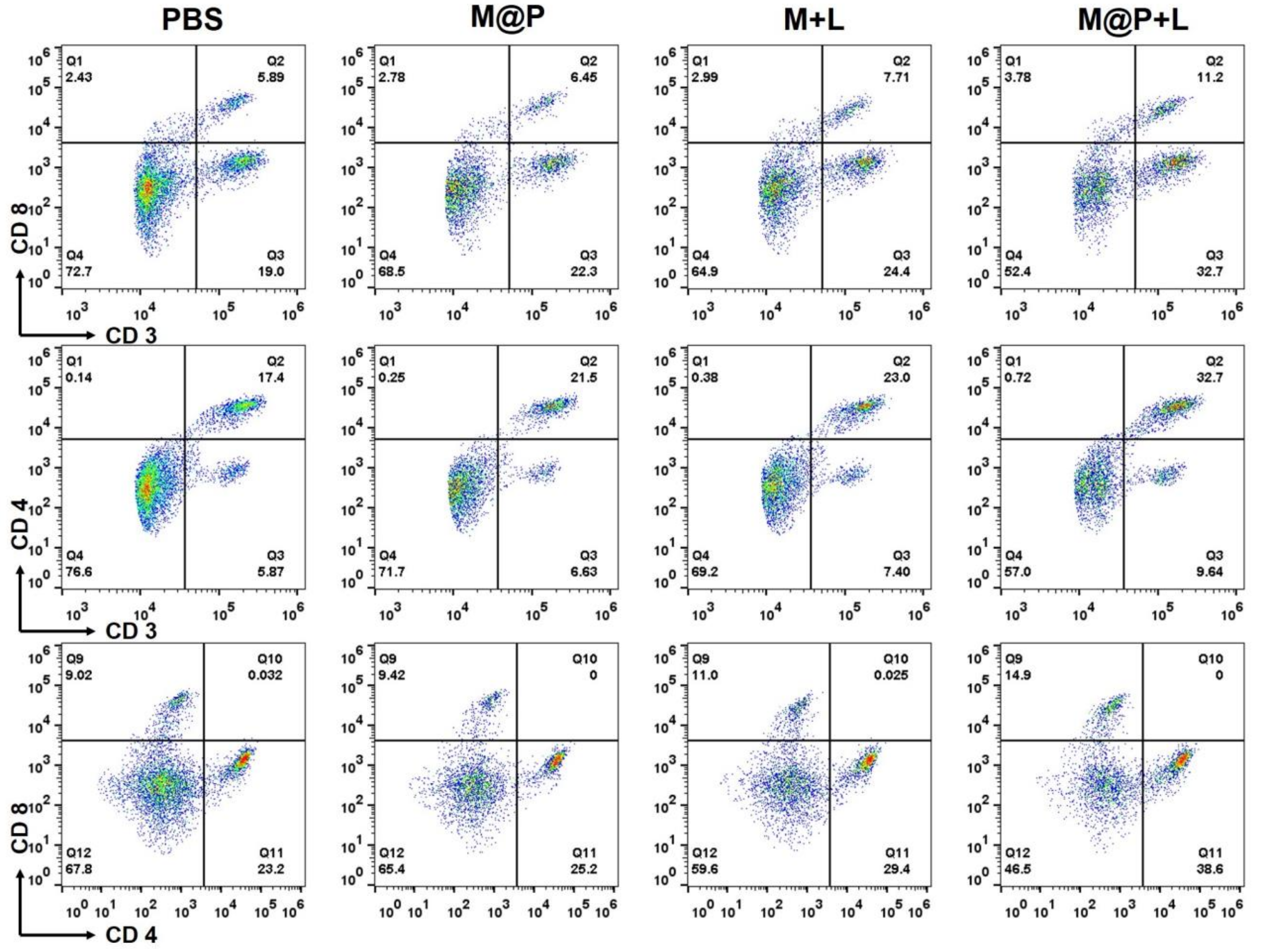

Fig. S36. Flow cytometry analysis of T lymphocytes ($CD8^+$ T cells and $CD4^+$ T cells gated on $CD3^+$ T cells) in serum.

Reference

1. Tang, Y. Q. et al. Pyroptosis-mediated synergistic photodynamic and photothermal immunotherapy enabled by a tumor-membrane-targeted photosensitive dimer. *Advanced Materials* **35**, 2300232 (2023).
